# Second-Generation Light-Fueled Supramolecular Pump

**DOI:** 10.1021/jacs.1c06027

**Published:** 2021-07-20

**Authors:** Martina Canton, Jessica Groppi, Lorenzo Casimiro, Stefano Corra, Massimo Baroncini, Serena Silvi, Alberto Credi

**Affiliations:** † CLAN-Center for Light Activated Nanostructures, ISOF-CNR, Via Gobetti 101, 40129 Bologna, Italy; ‡ Dipartimento di Chimica Industriale “Toso Montanari”, Università di Bologna, Viale Risorgimento 4, 40136 Bologna, Italy; § Dipartimento di Scienze e Tecnologie Agro-Alimentari, Università di Bologna, Viale Fanin 44, 40127 Bologna, Italy; ∥ Dipartimento di Chimica “G. Ciamician”, Università di Bologna, Via Selmi 2, 40126 Bologna, Italy

## Abstract

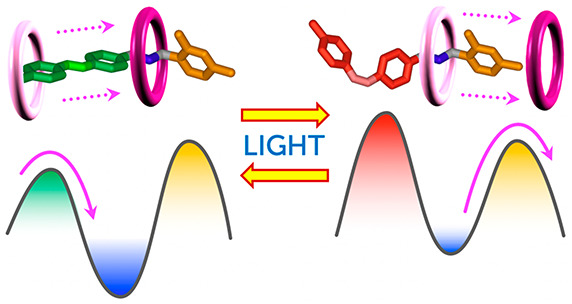

We describe the modular design of a pseudorotaxane-based supramolecular pump and its photochemically driven autonomous nonequilibrium operation in a dissipative regime. These properties derive from careful engineering of the energy maxima and minima along the threading coordinate and their light-triggered modulation. Unlike its precursor, this second-generation system is amenable to functionalization for integration into more complex devices.

Molecular pumps^1,2^ are nanoscale machines^3−6^ that can transport molecular or ionic substrates along a specific direction using an energy supply. While in channels the substrate moves down a concentration gradient, molecular pumps directionally transfer the substrate without a gradient or even against it.^7,8^ Hence, pumps working in solution can be used to move ions or molecules in a controlled way and to generate concentration differences between compartments separated by a membrane.^7,9^ In other words, these devices could regulate the presence of chemical species as well as transduce and store chemical energy in compartmentalized solutions.^10^ For these reasons, molecular pumps are essential elements of the set of biomolecular motors that sustain the life of organisms.^7,8,11^

Despite the relevance of molecular pumps in nature, very few examples of artificial counterparts have been described to date.^12−17^ All of them rely on pseudorotaxanes, i.e., supramolecular complexes consisting of an acyclic molecule (the axle) threaded through a macrocycle (the ring) and stabilized by non-covalent intermolecular interactions (Figure 1a).^18^ These pumps typically operate by an energy ratchet mechanism (Figure 1b).^5^ The axle, because of its nonsymmetric structure, has a kinetically preferred threading direction; once the pseudorotaxane is formed (i.e., equilibrium is reached), an applied energetic stimulus raises the potential energy minimum, thus creating a nonequilibrium complex. If also the relative height of the threading barriers is modified, the system will move toward a new equilibrium by directional dethreading. The stimulus-induced reset of the initial conditions closes the pumping cycle. Clearly, such a design requires careful engineering of the ring–axle intermolecular interactions and the ability to modulate them upon external chemical or physical inputs.^19^

**Figure 1:**
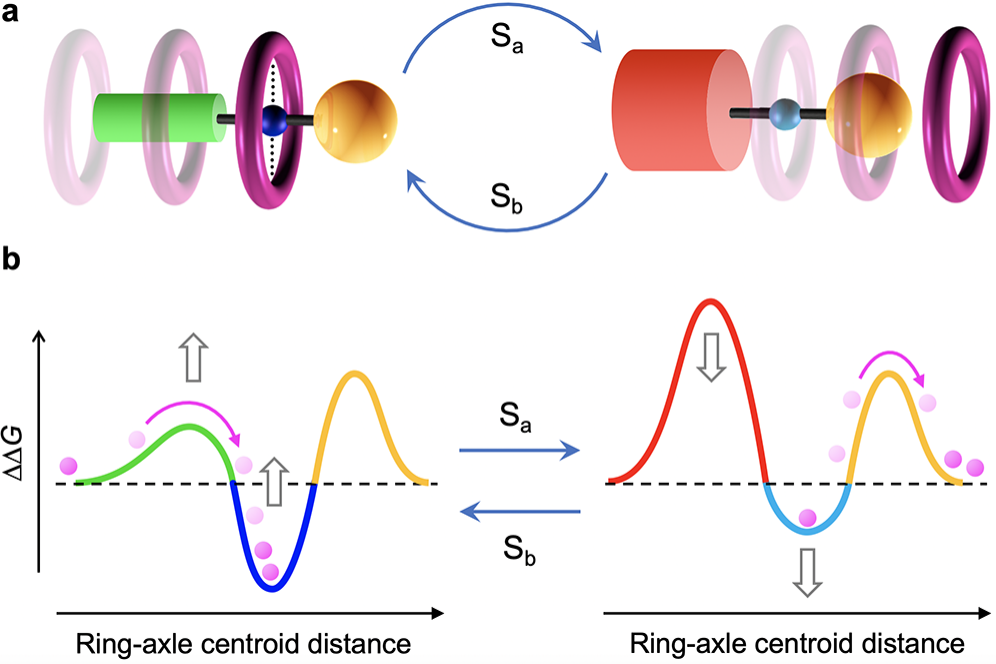
(a) A pseudorotaxane-based molecular pump operated by inputs S_a_ and S_b_. In the present system, S_a_ = S_b_ = *h*ν. (b) Simplified energy diagram showing the ratchet mechanism responsible for directional motion.

Stoddart and co-workers reported a series of redox-driven molecular pumps^14,15^ and utilized them to generate nonequilibrium poly(pseudo)rotaxanes.^20,21^ This outstanding work highlights the potential of threaded complexes to make novel molecular-based devices and materials. These pumps, however, cannot process the energy input in an autonomous fashion: the reduction and oxidation stimuli must be applied in a sequence by an operator (or an external device having the same function) with a frequency that depends on the motion kinetics.^22^ Although the system can be driven progressively away from equilibrium by repeating the input sequence, this limitation makes such a task intrinsically slow and renders extensive cycling impractical. It should be recalled that all biomolecular motors are autonomous; in most instances, they operate by catalytically hydrolyzing ATP in a constant environment.^11^

We previously described a pseudorotaxane-based molecular pump that works autonomously under constant light irradiation in mild conditions.^13^ The system consists of the 2,3-dinaphtho[24]crown-8 ether **1** and axle **2**^+^ (Chart 1) comprising a photoswitchable azobenzene gate, a secondary ammonium recognition site, and a non-photoactive cyclopentyl pseudostopper. Key for the implementation of the ratchet mechanism depicted in Figure 1b with components **1** and **2**^+^ is the fact that the *E* → *Z* photoisomerization of the azobenzene gate causes both a destabilization of the complex and an increase of the threading barrier at the azobenzene end, which becomes larger than that at the pseudostopper end.^23^ The fact that the threading barrier at the pseudostopper end lies between those at the *E*- and *Z*-azobenzene ends enables the directionality at the basis of the pump operation. Autonomous cycling away from equilibrium can be achieved by light irradiation because both azobenzene configurational isomers are photoreactive and possess common absorption regions.^24^

**Chart 1:**
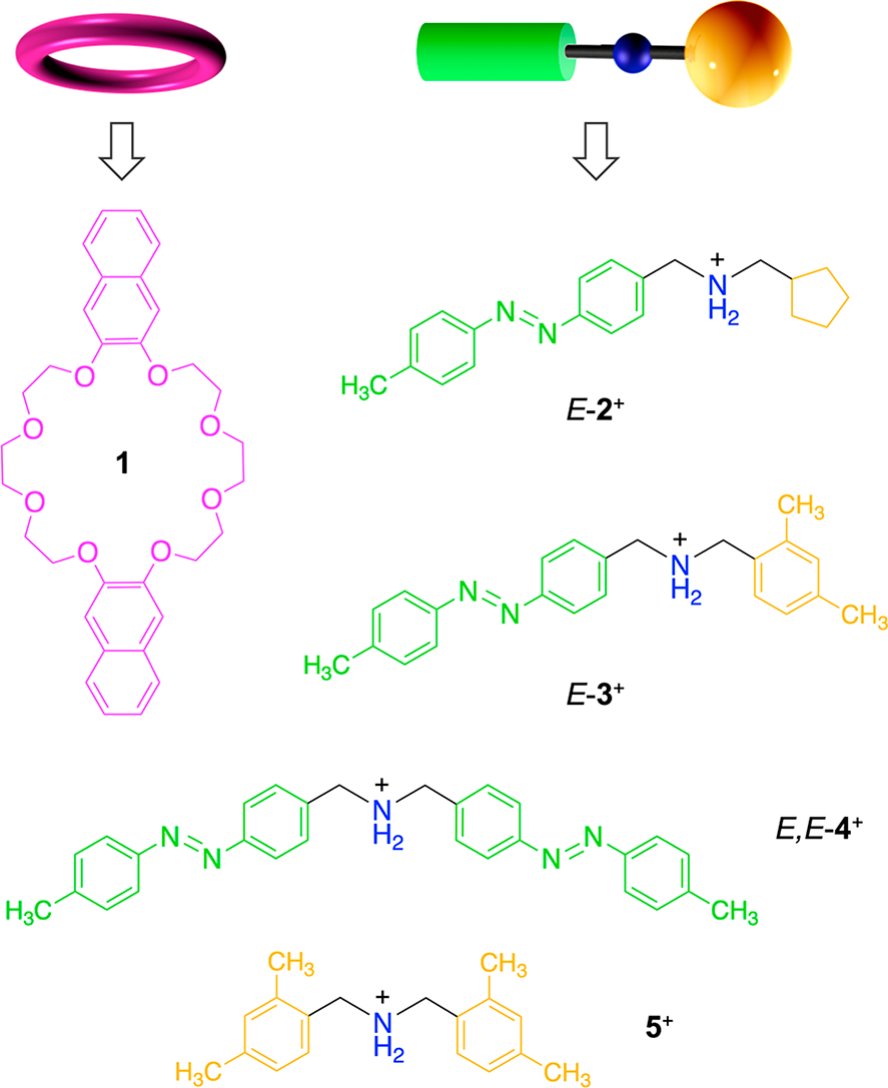
Structural Formulas of Macrocycle **1**, First-Generation Axle **2**^+^, Second-Generation Axle **3**^+^ (The Object of This Work), and Symmetric Model Compounds **4**^+^ and **5**^+^a

a For all of the axles, the counterion is PF_6_^–^.

To integrate a supramolecular pump into more sophisticated functional devices, its axle component should be amenable to connection with other parts.^20^ Unfortunately, the cyclopentyl moiety of **2**^+^, being a cycloalkane, is hard to functionalize in a clean and controlled way. Moreover, the presence of two substituents on the cyclopentyl ring would lead to trans and cis stereoisomers that could exhibit different threading behaviors. These issues hamper the versatility of the device and render it unsuitable as a general-purpose pump module.

In the attempt to replace the cyclopentyl unit with a substituted phenyl moiety while maintaining the successful design of **2**^+^, we recently performed a systematic investigation^25^ of the threading kinetics of dibenzylammonium-type axles with dibenzo[24]crown-8, which has a cavity identical to that of **1**. This study revealed that a 2,4-dimethylphenyl moiety exhibits threading behavior qualitatively similar to that of the cyclopentyl group under the employed experimental conditions (CH_2_Cl_2_, room temperature), i.e., its passage through the cavity of the macrocycle occurs at a rate that is intermediate between those of the *E*- and *Z*-azobenzene units. Hence, the 2,4-dimethylphenyl unit possesses the requirements to act as a pseudostopper according to the mechanism shown in Figure 1 combined with the rich and well-known reactivity of aromatic rings. Here we present the new photoactive axle **3**^+^ (Chart 1), and the thermodynamic and kinetic properties of its self-assembly with macrocycle **1** in the dark and under light irradiation. The objective is to verify whether the **1**–**3**^+^ ensemble functions as a photochemically driven autonomous molecular pump and to observe its dissipative nonequilibrium operation under light irradiation.

Both the *E* and *Z* isomers of **3**^+^ form threaded 1:1 complexes with **1** in CH_2_Cl_2_, as demonstrated by ^1^H NMR and UV–vis absorption and luminescence spectroscopies (see the Supporting Information (SI)). In particular, the fluorescence of **1** (λ_max_ = 345 nm) is quenched in the complex with either isomer of the axle. The stability constants (*K*), determined by spectrofluorimetric titrations (Figure 2), and the threading rate constants (*k*_in_), measured by monitoring the time-dependent absorbance changes after mixing of the components (Figure 2, insets), are summarized in Table 1 together with the calculated dethreading rate constants (*k*_out_). The data for the symmetric model compounds **4**^+^ and **5**^+^ (Chart 1) are also reported for comparison.

**Table 1:** Thermodynamic and Kinetic Constants (CH_2_Cl_2_, 293 K)

complex	*K*^a^ (M^–1^)	*k*_in_^b^ (M^–1^ s^–1^)	*k*_out_^c^ (s^–1^)
[*E*-**3**⊂**1**]^+^	5 × 10^6^	45	9 × 10^–6^
[*Z*-**3**⊂**1**]^+^^d^	3 × 10^5^	5.5	1.8 × 10^–5^
[*E*,*E*-**4**⊂**1**]^+^^e^	>10^7^	55	<5.5 × 10^–6^
[*Z*,*Z*-**4**⊂**1**]^+^^d^^,^^e^	–f	3.9 × 10^–2^	–f
[**5**⊂DB24C8]^+^^g^	1.7 × 10^5^	12	7 × 10^–5^

a Determined by UV–vis titrations.

b Determined by time-resolved UV–vis absorption spectroscopy.

c Calculated as *k*_out_ = *k*_in_/*K*.

d Obtained by exhaustive irradiation of the *E* isomer at 365 nm.

e From ref (13).

f Not determined because the formation of the complex is slower than the thermal *Z* → *E* isomerization.

g From ref (25).

**Figure 2:**
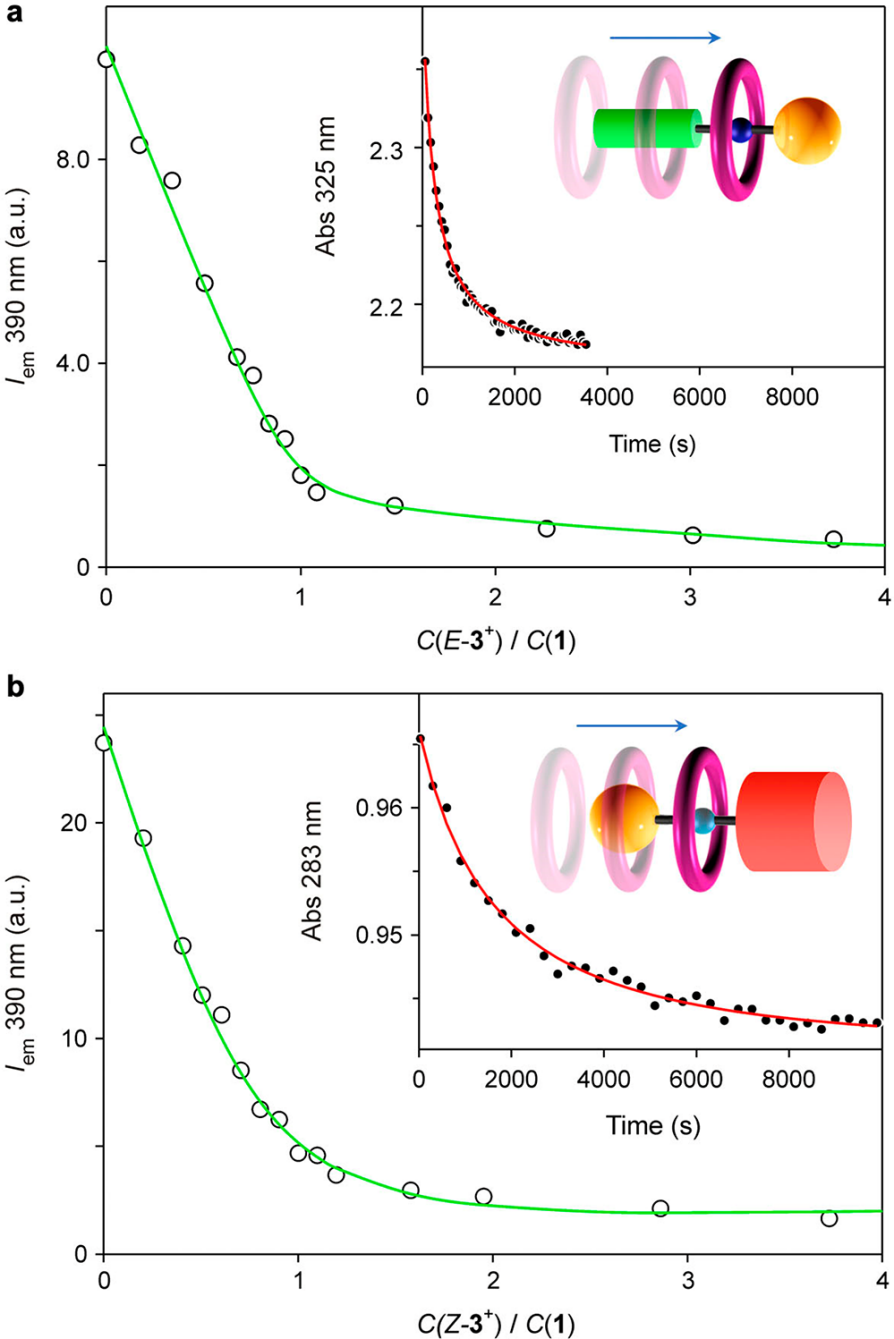
Fluorescence titration curves (λ_ex_ = 281 nm, λ_em_ = 390 nm) observed upon addition of (a) *E*-**3**^+^ or (b) *Z*-**3**^+^ to 50 μM **1**. The lines are best fits to a 1:1 binding model. The insets show the time-dependent absorption changes observed upon 1:1 mixing of **1** and (a) *E*-**3**^+^ or (b) *Z*-**3**^+^. Concentration after mixing: 200 μM. The lines are best fits to a kinetic model comprising a second-order forward reaction and a first-order backward reaction. CH_2_Cl_2_, 293 K.

The *Z* isomers of the axles and their complexes were obtained by exhaustive irradiation of the corresponding *E* forms at 365 nm, which led to photostationary states (PSSs) containing 96% *Z*-form. The *E* → *Z* and *Z* → *E* photoisomerization quantum yields of **3**^+^ are in line with those of azobenzene and are not affected by complexation with **1**. The half-life of *Z*-**3**^+^ is about 30 h at 293 K and becomes 64 h when the axle is encircled by the ring.

In line with observations made on axle **2**^+^,^13^ the complex of **1** with *E*-**3**^+^ is more stable than that with *Z*-**3**^+^, most likely because of π stacking between the naphthalene moieties of **1** and the planar *E*-azobenzene unit of the axle.^26^ However, while the stability constant of [*E*-**3**⊂**1**]^+^ (*K*_*E*_) is larger than that of [*E*-**2**⊂**1**]^+^, possibly because of additional π stacking involving the dimethylphenyl moiety, the stability constants of the corresponding *Z* complexes (*K*_*Z*_) are in the opposite order. Presumably, the *Z*-azobenzene moiety not only is unable to interact with the macrocycle but also disturbs its π stacking with the pseudostopper unit. Because of this phenomenon, the *K*_*E*_/*K*_*Z*_ ratio, whose deviation from unity is key to generate a photoinduced dissipative nonequilibrium state,^13,27,28^ increases from 3.7 for **2**^+^ to 17 for **3**^+^.

The threading rate constant of *E*-**3**^+^ is slightly smaller than that of the symmetric azobenzene model *E*,*E*-**4**^+^ and about 8 times larger than that of the symmetric pseudostopper model **5**^+^ (Table 1). In contrast, the threading rate constant of *Z*-**3**^+^ is roughly half that of **5**^+^ and 140 times larger than that of *Z*,*Z*-**4**^+^. Considering statistical factors when symmetric axles (in which two extremities are available for threading) are compared with nonsymmetric ones, these results clearly indicate that *E*-**3**^+^ enters ring **1** preferentially (∼82%) with the *E*-azobenzene extremity, whereas threading of *Z*-**3**^+^ occurs exclusively (>99%) from its dimethylphenyl terminus. Taken together, these data confirm that light irradiation causes ratcheting of the energy profile (Figure 1b; a more realistic representation based on the experimentally determined energy values is shown in Figure 3a) that imparts directionality to the Brownian motion of the ring relative to the axle.

**Figure 3:**
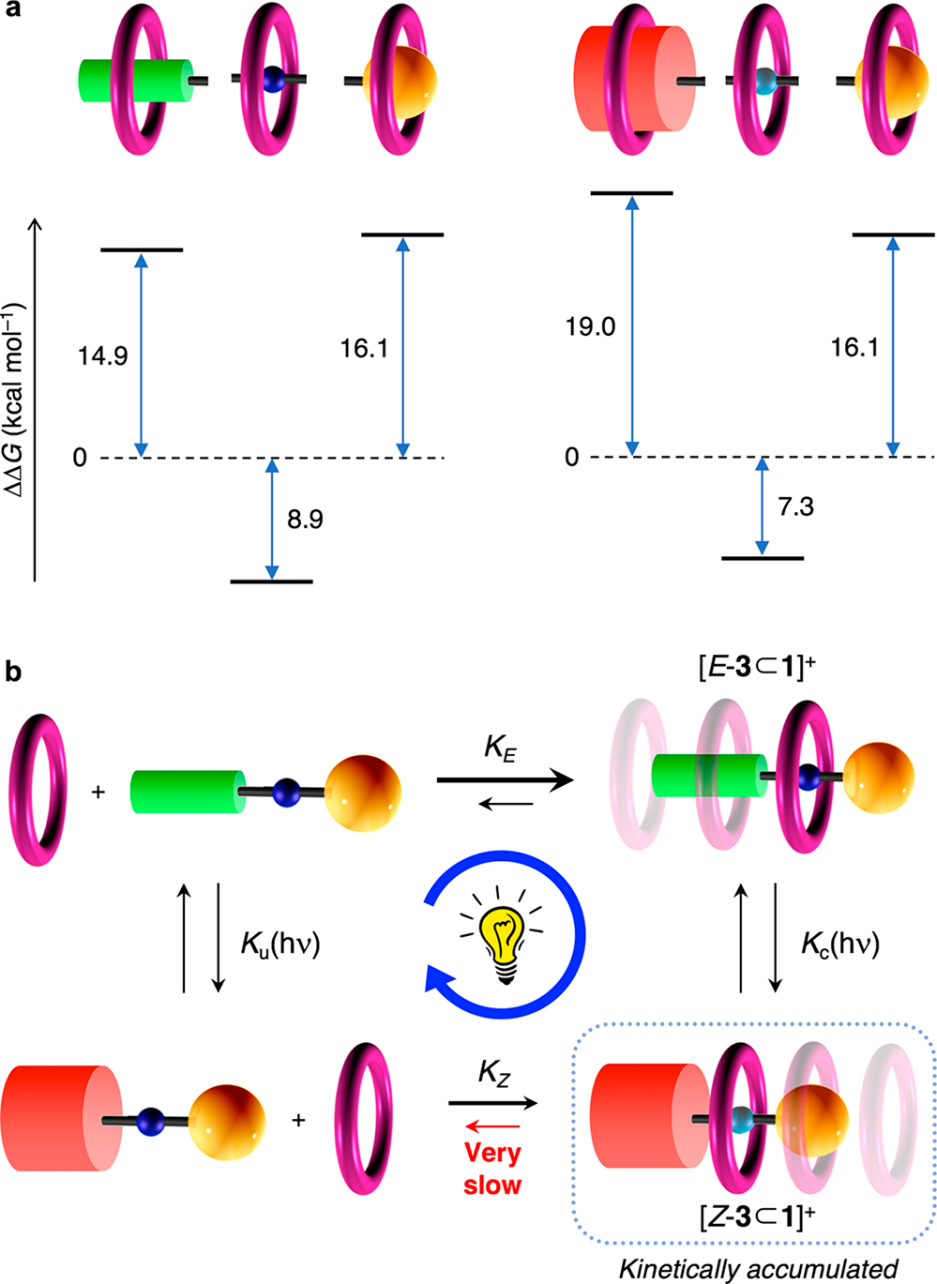
(a) Free energy levels, with respect to the dissociated components, along the threading coordinate of **1** with (left) *E*-**3**^+^ and (right) *Z*-**3**^+^ in CH_2_Cl_2_ at 293 K. (b) Reaction network representing the operation of the motor. Horizontal and vertical processes are the self-assembly and photoisomerization reactions, respectively.

Another significant aspect of this system, shared with its precursor,^13^ is its ability to use light energy to operate in a dissipative regime away from equilibrium.^28−30^ Unlike kinetically trapped or metastable nonequilibrium states, a dissipative state can exist only if energy is continuously supplied.^30−33^ The closed reaction network shown in Figure 3b consists of chemical reactions, which are subject to microscopic reversibility, and photochemical reactions, which are not. The constants *K*_u_(*h*ν) and *K*_c_(*h*ν) of the photochemical branches can be taken as the ratios of the concentrations of the respective products and reactants at the PSS. Under our conditions, the steady-state compositions of the uncomplexed and complexed axles upon 365 nm irradiation are the same, that is, *K*_u_(*h*ν) = *K*_c_(*h*ν). Since *K*_*E*_ > *K*_*Z*_, detailed balance is not fulfilled, and the system goes through the cycle following preferentially a clockwise path (Figure 3b); at the steady state, the net rates of the individual processes in the cycle are equal but nonzero. For a thorough mechanistic description, see refs (13), (27), and (28).

We employed ^1^H NMR spectroscopy to measure the concentrations under continuous light irradiation,^34^ which was performed by introducing an optical fiber into the NMR probe (see the SI).^35^ A distinctive feature of the dissipative nonequilibrium condition obtained upon photochemical cycling is the accumulation of [*Z*-**3**⊂**1**]^+^. This species is less stable than [*E*-**3**⊂**1**]^+^, but its disassembly is the slowest step of the cycle, making it a kinetic sink (Figure 3b). If the system is brought to the PSS (365 nm) rapidly with respect to dethreading of the *Z* complex, a nonequilibrium concentration of [*Z*-**3**⊂**1**]^+^ is generated (Figure 4). In the dark, such a concentration decreases by ca. 15% in a few hours because of dethreading from the pseudostopper end (Figure 4a, pale trace). It should be noted that the thermal *Z* → *E* isomerization is almost negligible on this time scale (dashed line). Conversely, when the light is kept on, the concentration of [*Z*-**3**⊂**1**]^+^ does not decrease; rather, it slightly increases until a constant value is reached after ca. 7 h of irradiation (Figure 4a, vivid trace). The transition from the dissipative to the kinetically trapped nonequilibrium regime can be clearly seen in Figure 4b. The [*Z*-**3**⊂**1**]^+^ complex disappears in the dark because of dethreading (faster decrease) and thermal *Z* → *E* azobenzene isomerization (slower decrease); the latter process is mirrored by the slow increase of [*E*-**3**⊂**1**]^+^.

**Figure 4:**
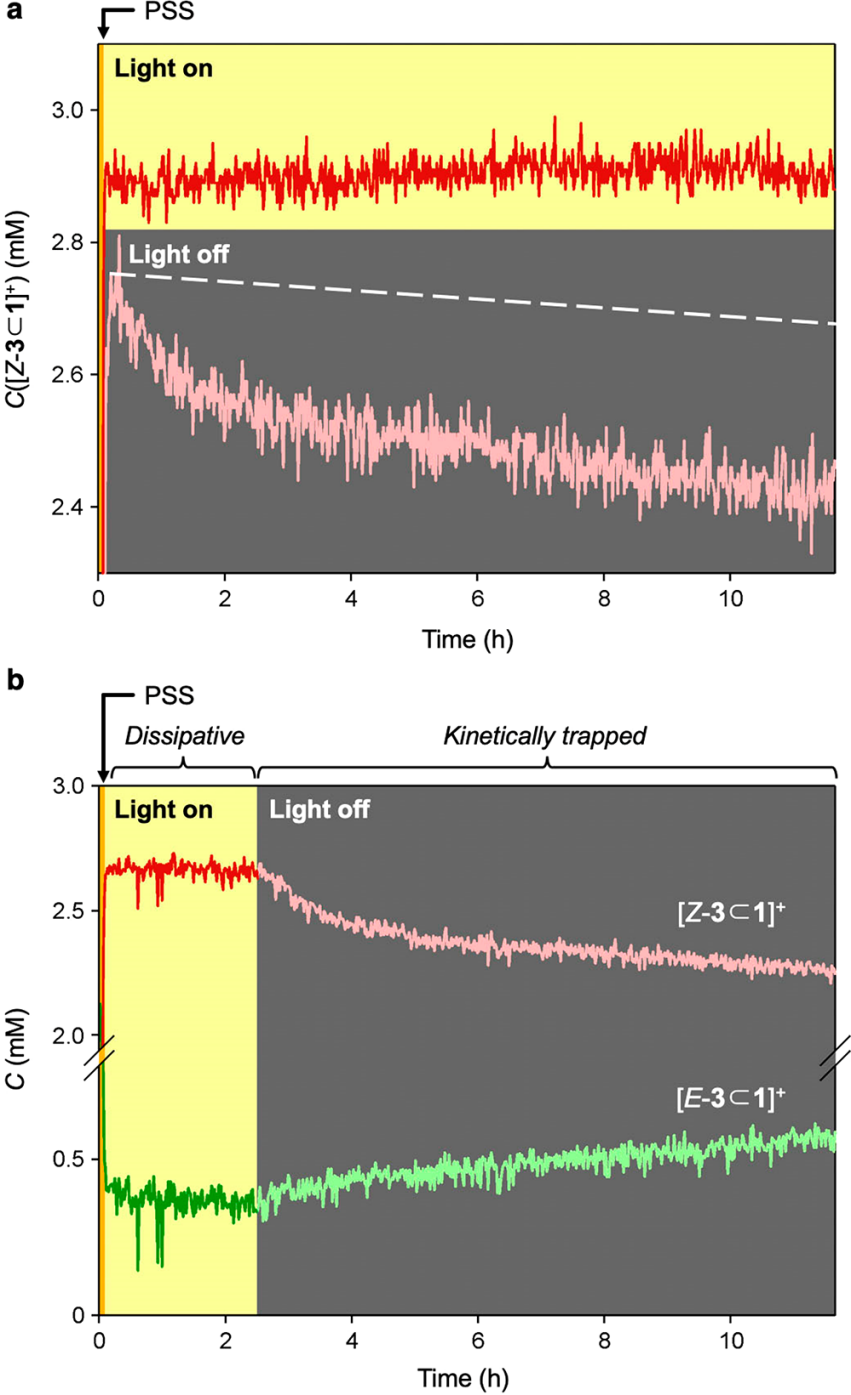
Time-dependent concentration changes of (a) [*Z*-**3**⊂**1**]^+^ and both (b) both the *E* and *Z* complexes detected on a 1:1 mixture of **1** and *E*-**3**^+^ (5 mM) under 365 nm irradiation and dark conditions. The PSS is reached within the initial 300 s (orange bar). Traces in vivid and pale colors refer to concentrations measured under irradiation (yellow background) and in the dark (gray background), respectively. The dashed line in (a) shows the calculated thermal decay of the *Z* form. CD_2_Cl_2_/CD_3_CN 1:1, 298 K.

In conclusion, we have described an autonomous light-driven supramolecular pump in which the ring exit extremity of the axle bears a substituted phenyl moiety. Unlike its progenitor,^13,28^ the present axle is amenable to elongation at the exit side—an important requirement for insertion of the motor into more sophisticated molecular devices. We have provided direct evidence for photoinduced operation of the system away from equilibrium in a dissipative regime. Our results highlight the modularity and versatility of this family of supramolecular pumps, which together with its minimalist design and synthetic accessibility make it a unique platform for studying and exploiting light-fueled nonequilibrium phenomena to create dynamic systems.^36^

## Supporting Information Available

Methods, synthetic details, and spectroscopic and photochemical data (PDF)
