# Supplementary material for: Second-Generation Light-Fueled Supramolecular Pump
Source: J Am Chem Soc. 2021 Jul 20;143(29):10890–4. doi: 10.1021/jacs.1c06027 (PMC8323096; doi:10.1021/jacs.1c06027)
Supplement: Supplementary file 1 — ja1c06027_si_001.pdf [file ja1c06027_si_001.pdf]

Second Generation Light-Fueled Supramolecular Pump

Martina Canton, Jessica Groppi, Lorenzo Casimiro, Stefano Corra, Massimo Baroncini, Serena Silvi, and Alberto Credi

CLAN-Center for Light Activated Nanostructures, ISOF-CNR, Via Gobetti 101, 40129 Bologna, Italy

Dipartimento di Chimica Industriale "Toso Montanari", Università di Bologna, Viale Risorgimento 4, 40136 Bologna, Italy

Dipartimento di Scienze e Tecnologie Agro-Alimentari, Università di Bologna, Viale Fanin 44, 40127 Bologna, Italy

Dipartimento di Chimica "G. Ciamician", Università di Bologna, Via Selmi 2, 40126 Bologna, Italy

SUPPORTING INFORMATION

Table of Contents

1. Materials and Methods S2
2. Synthetic Procedures S4
3. ^{1}H NMR Spectroscopy S7
4. UV-Visible Spectroscopy S18
5. References S25

1. Materials and Methods

General. *p*-nitrobenzylamine,[1] nitrosotoluene,[2] dinaphto[24]crown-8,[3] symmetric model compounds *E,E*-**4**$^{+}$,[4] and **5**$^{+}$ [5] were synthesized according to literature procedures. All reagents and chemicals were purchased from Sigma-Aldrich or VWR international and used as received unless otherwise stated. Solvents were dried according to literature procedures. Hydrogenation reactions were performed using a ThalesNano H-cube flow reactor on prepacked catalyst cartridges CatCart purchased from Sigma-Aldrich. Flash column chromatography was performed using Sigma Aldrich Silica 40 (230-400 mesh size or 40-63 mm) as the stationary phase. Thin layer chromatography was performed on TLC Silica gel 60 F254 coated aluminium plates from Merck.

^{1}H NMR Spectroscopy. NMR spectra were recorded on an Agilent DD2 spectrometer operating at 500 MHz. Chemical shifts are quoted in part per million (ppm) relative to tetramethylsilane using the residual solvent peak as a reference standard and all coupling constants (*J*) are expressed in Hertz (Hz).

UV-Visible Spectroscopy. Absorption and luminescence spectra were performed with a Cary 300 and with a Cary 50Bio (Varian) spectrophotometers and a FS5 Edinburgh spectrofluorimeter, respectively, on air equilibrated CH_2Cl_2 solutions at room temperature (293 K). Regular 1-cm spectrofluorimetric quartz cells were used for the spectroscopic experiments. Specific spectrophotometric cells endowed with two compartments separated by a quartz wall were employed for a precise determination of the sum of the absorption spectra of the separated reactants (unmixed solutions) and the absorption spectrum of the product (mixed solutions). The concentration of the solutions was comprised between 1×10^{-5} and 1×10^{-3} M.

Stability constants were determined from titration experiments performed by monitoring the luminescence intensity of **1** (λ_{exc} = 281nm) upon addition of small aliquots of a concentrated solution of the axle (mM) to a dilute solution (typically, 50 μM) of **1** in CH_2Cl_2 at 293 K. To minimize inner filter effects, the emission intensity was measured on an isosbestic point (390 nm). The constants were calculated by means of the SPECFIT fitting program[6] according to a 1:1 binding model (equation S1). The reported values are an average of at least two independent experiments.

$\mathbf{1}$ + axle $\leftrightarrows$ [$\mathbf{1}\supset$axle] (S1)

Reaction kinetic profiles were collected on air equilibrated CH_2Cl_2 (1×10^{-4} M) solutions at 293 K. Threading processes were monitored by following the time-dependent spectroscopic changes observed after addition of a concentrated (typically mM) solution of the ring to a more dilute axle solution. Thermal $Z\rightarrow E$ isomerization reactions were performed at 293 K in the dark, monitoring the time-dependent absorption changes. The data were elaborated by means of the SPECFIT fitting program. Threading processes were modeled according to a mixed-order model, that is, second order (threading) and first order (dethreading) opposing reactions (equation S1). Dethreading processes and thermal $Z\rightarrow E$ isomerization reactions were modelled according to first order kinetics.

The threading efficiencies for either extremity of the non-symmetric axle $\mathbf{3}^+$ were estimated according to equations S2-S3:

$$\eta_{E/Z\text{-azo}} = k_{E/Z\text{-azo}} / (k_{E/Z\text{-azo}} + k_{ps}) \quad (S2) \qquad \eta_{ps} = k_{ps} / (k_{E/Z\text{-azo}} + k_{ps}) \quad (S3)$$

in which $k_{E/Z\text{-azo}}$ is the threading rate constant for the azobenzene extremity in the relevant configuration (*E* for *E*-$\mathbf{3}^+$ and *Z* for *Z*-$\mathbf{3}^+$), and k_{ps} is the threading rate constant for the 2,4-dimethylphenyl pseudostopper terminus. The symmetric axles shown in Chart 1 were taken as models for threading on different extremities, taking into account that in these compounds two identical ends are available for threading. Hence, $k_{E\text{-azo}}$ in the case of *E*-$\mathbf{3}^+$ was estimated to be k_{in} of *E,E*-$\mathbf{4}^+$ divided by 2; similarly, $k_{Z\text{-azo}}$ for *Z*-$\mathbf{3}^+$ was taken as the half of k_{in} of *Z,Z*-$\mathbf{4}^+$. For both isomers of $\mathbf{3}^+$, k_{ps} was taken as $k_{in}/2$ of $\mathbf{5}^+$.

Experimental errors: wavelengths, ±1 nm; molar absorption coefficients, ±10%; log*K*, ±7%; rate constants, ±20%.

Photochemical reactions. Photochemical processes were monitored in CH_2Cl_2 solutions at room temperature (ca. 293 K). The irradiations were performed by employing a Hanau Q400 medium pressure Hg lamp (150 W). The selection of the chosen irradiation wavelength (365 or 436 nm) was achieved by means of a suitable interference filter. The number of incident photons was

calculated by ferrioxalate actinometry in the micro version.[7] The flux of incidents photons was typically 2.1×10^{-7} Einstein min^{-1} at 365 nm, and 2.4×10^{-7} Einstein min^{-1} at 436 nm.

^1H NMR photochemical isomerization experiments were performed in air equilibrated solutions in a solvent mixture composed by CD_3CN/CD_2Cl_2 in 1:1 ratio at 298 K. Neat dichloromethane could not be used because the very strong association at the concentrations employed in the NMR experiments prevents the detection of the uncomplexed components. On the other hand, macrocycle **1** is poorly soluble in neat acetonitrile. The employed dichloromethane/acetonitrile mixture afforded complete solubilization of the compounds and allowed the monitoring of both complexed and uncomplexed species during the ^1H NMR experiments. Irradiation of NMR samples within the spectrometer probe was performed with a setup adapted from the one described by Gschwind and coworkers.[8] The irradiation system consists of a Prizmatix UHP-T-365-SR LED Illuminator (1.5 W, λ_{max}= 369 nm, FWHM, 15.56 nm) equipped with FCA-SMA adaptor for the optical fiber. POF Polymer Optical Fiber (core 1500 μm, 4 m) with a SMA connector on one end was also purchased from Prizmatix; the other end of the optical fiber was scraped to remove the protective coating and was submerged into the solution within the NMR tube to be irradiated.

Experimental errors: photoreaction quantum yields, ±10%.

2. Synthetic procedures

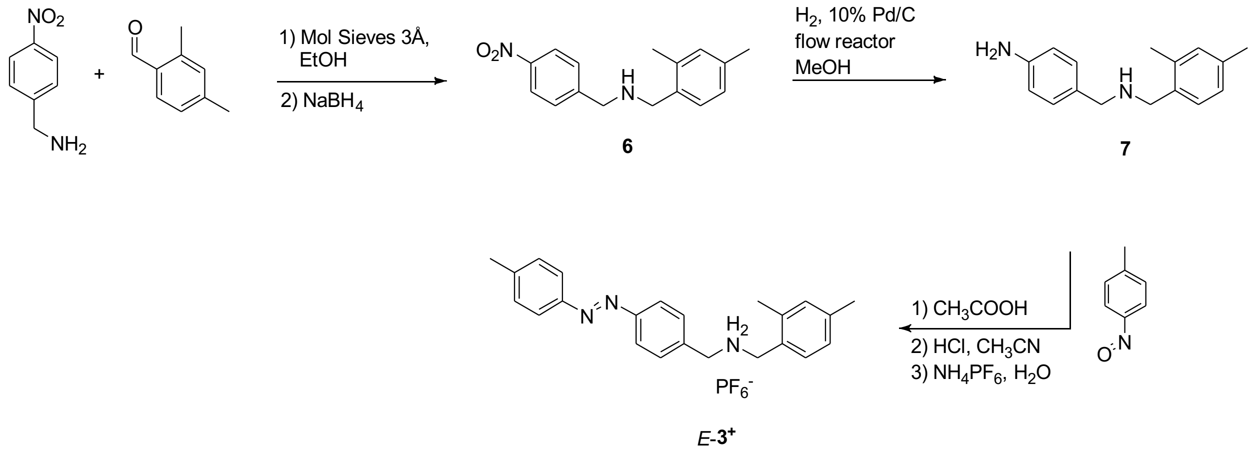

Scheme S1: Synthetic steps toward compound E-**3**$^+$•PF_6^-.

N-(2,4-dimethylbenzyl)-1-(4-nitrophenyl)methanamine (6)

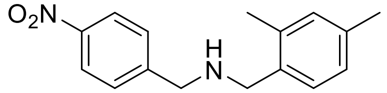

p-nitrobenzylamine hydrochloride (0.69 g, 3.6 mmol) was suspended in dry EtOH (30 ml) and NaOH (0.17 g, 4.2 mmol) dissolved in dry EtOH was added to deprotonate the amine. The solution was stirred for 15 minutes at room temperature. 2,4-dimethylbenzaldehyde (0.46 g, 3.4 mmol) was added to the solution followed by 3 Å molecular sieves and the mixture was stirred at room temperature for 2 hours. $NaBH_4$ (0.13 g, 3.5 mmol) was added portion-wise and the mixture was stirred until the TLC (Hex : EtOAc = 8 : 2) showed complete consumption of the starting aldehyde and the corresponding imine (about 3 hours). The reaction solvent was removed, and the residue obtained was dissolved with H_2O (100 ml) and EtOAc (50 ml) and the aqueous phase was extracted with EtOAc (3 x 50 ml) to recover the crude product. The combined organic layers were dried over Na_2SO_4 and the solvent removed. The product was purified by silica column chromatography (eluent: Hex : EtOAc = 7 : 3). The product was a yellow solid obtained in 54 % (0.5 g) yield. **^{1}H-NMR (500 MHz, $CDCl_3$, 298 K):** *δ (ppm)* 8.18 (d, *J* = 8.00 Hz, 2H, Ar-H), 7.54 (d, *J* = 8.05 Hz, 2H, Ar-H), 7.17 (d, *J* = 7.95 Hz, 1H, Ar-H), 6.98-7.01 (m, 2H, Ar-H), 3.94 (s, 2H, CH_2), 3.75 (s, 2H, CH_2), 2.31 (s, 6H, CH_3). **^{13}C-NMR (125 MHz, $CDCl_3$, 298 K):** *δ (ppm)* 148.3, 147.2, 139.9, 128.8, 128.7, 128.2, 127.4, 123.8, 53.4, 52.5, 20.2, 18.3.

4-(((2,4-dimethylbenzyl)amino)methyl)aniline (7)

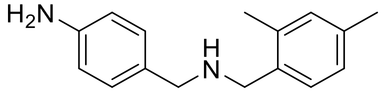

Compound **6** (0.5 g, 1.9 mmol) was dissolved in MeOH (50 ml) and the solution obtained was allowed to continuously flow through a pretreated 10% Pd/C catalyst CatCart cartridge combined with a constant flow of H_2 in full mode. The parameters set on the flow reactor were flow rate 1 ml/min, temperature 25°C, inlet pressure 10 bar. The complete reduction of the starting material after one reduction cycle was confirmed by TLC (Hex : EtOAc = 7 : 3). The solvent was removed to obtain the product as a clear oil in 95 % (0.41 g) yield. **^{1}H-NMR (500 MHz, $CDCl_3$, 298 K):** *δ*

(ppm) 7.18 (d, *J* = 7.95 Hz, 1H, Ar-H), 7.14 (d, *J* = 8.05 Hz, 2H, Ar-H), 6.97 (m, 2H, Ar-H), 6.66 (d, *J* = 8.00 Hz, 2H, Ar-H), 3.73 (s, 4H, CH_2), 2.30 (s, 3H, CH_3), 2.29 (s, 3H, CH_3) **^{13}C-NMR (125 MHz, $CDCl_3$, 298 K):** *δ (ppm)* 145.4, 140.6, 130.5, 129.4, 128.5, 128.3, 127.0, 115.2, 53.2, 52.9, 20.1, 18.1.

(*E*)-N-(2,4-dimethylbenzyl)-1-(4-(p-tolyldiazenyl)phenyl)methanaminium hexafluorophosphate (*E*-3$^+$)

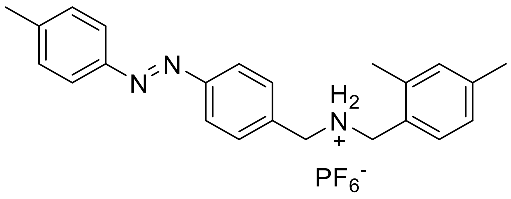

Nitrosotoluene (0.39 g, 3.2 mmol) and compound **7** (0.38 g, 1.6 mmol) were dissolved in CH_3COOH (10 ml) and left stirring in the dark overnight at room temperature. The solvent was removed and the solid obtained was dissolved in EtOAc (50 ml) and the organic phase was washed with $NaHCO_3$ (sat) (2x50 ml), then H_2O (50 ml) and brine (50 ml). The organic phase was dried over Na_2SO_4 and the solvent was removed. The product was purified by silica column chromatography to obtain the neutral derivative as an orange solid. This compound was converted into the final product by dissolving it in the minimum volume of CH_3CN and adding a few drops of concentrated HCl to precipitate the chloride salt. The anion was then exchanged to hexafluorophosphate by dissolving the compound in H_2O with a few drops of acetone and adding a saturated solution of NH_4PF_6 (aq). The product was an orange solid obtained in 50 % yield (0.39 g). **^{1}H-NMR (500 MHz, CD_2Cl_2, 298 K):** *δ (ppm)* 8.00 (d, *J* = 8.30 Hz, 2H, *Azo*Ar-H), 7.85 (d, *J* = 8.25 Hz, 2H, *Azo*Ar-H), 7.60 (d, *J* = 8.35 Hz, 2H, *Azo*Ar-H), 7.37 (d, *J* = 8.25 Hz, 2H, *Azo*Ar-H), 7.25 (d, *J* = 8.30 Hz, 1H, *PS*Ar-H), 7.09-7.12 (m, 2H, *PS*Ar-H), 4.31 (s, 2H, CH_2), 4.23 (s, 2H, CH_2), 2.47 (s, 3H, *Azo*CH_3), 2.32 (s, 3H, *PS*CH_3), 2.24 (s, 3H, *PS*CH_3) **^{13}C-NMR (125 MHz, CD_2Cl_2, 298 K):** *δ (ppm)* 153.2, 150.9, 142.6, 139.7, 137.5, 134.9, 132.0, 131.0, 130.6, 130.1, 127.7, 127.6, 123.6, 123.3, 50.7, 47.8, 21.6, 21.2, 18.9. **^{19}F-NMR (470 MHz, CD_2Cl_2, 298 K):** *δ (ppm)* -71.3 (d, $J_{F\text{-}P}$ = 713 Hz). **HRMS (ESI+):** m/z calc. for $C_{23}H_{26}N_3$ $[M]^+$: 345.2205, found: 345.2205.

3. NMR Spectroscopy

3.1. NMR characterization of compounds

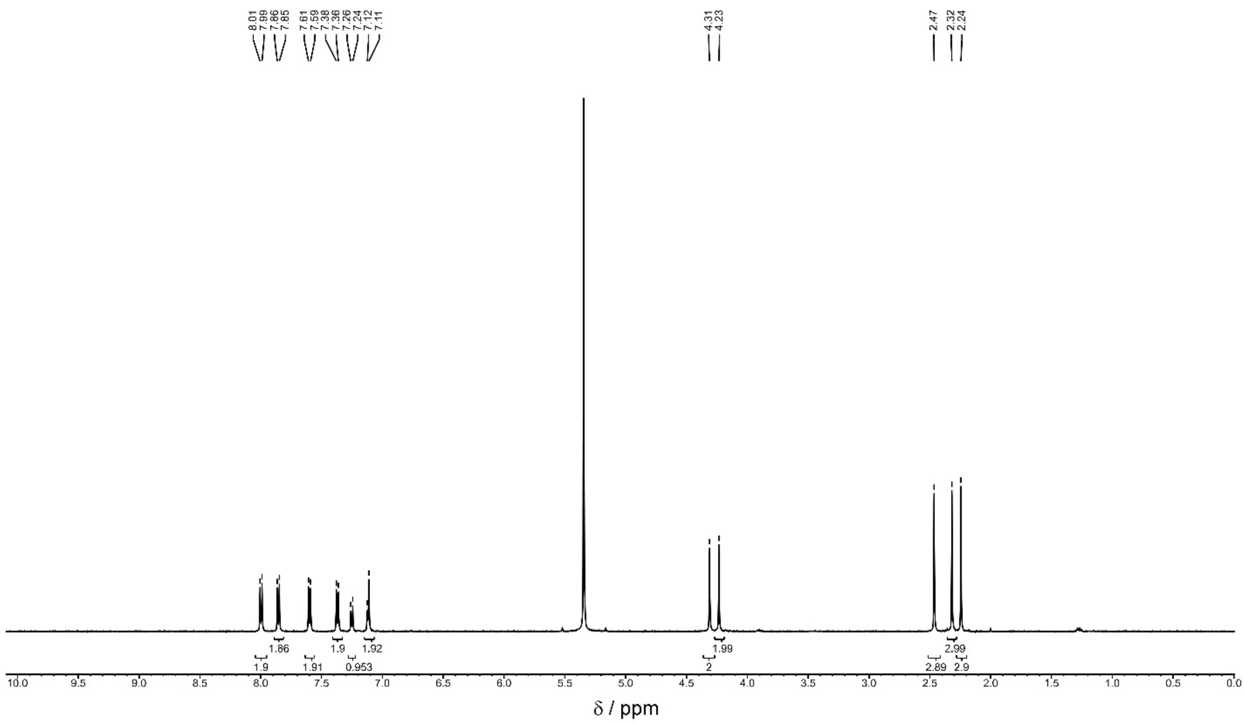

Figure S1: ^{1}H NMR Spectrum (500 MHz, CD_2Cl_2, 298 K) of compound *E*-**3**$^+$.

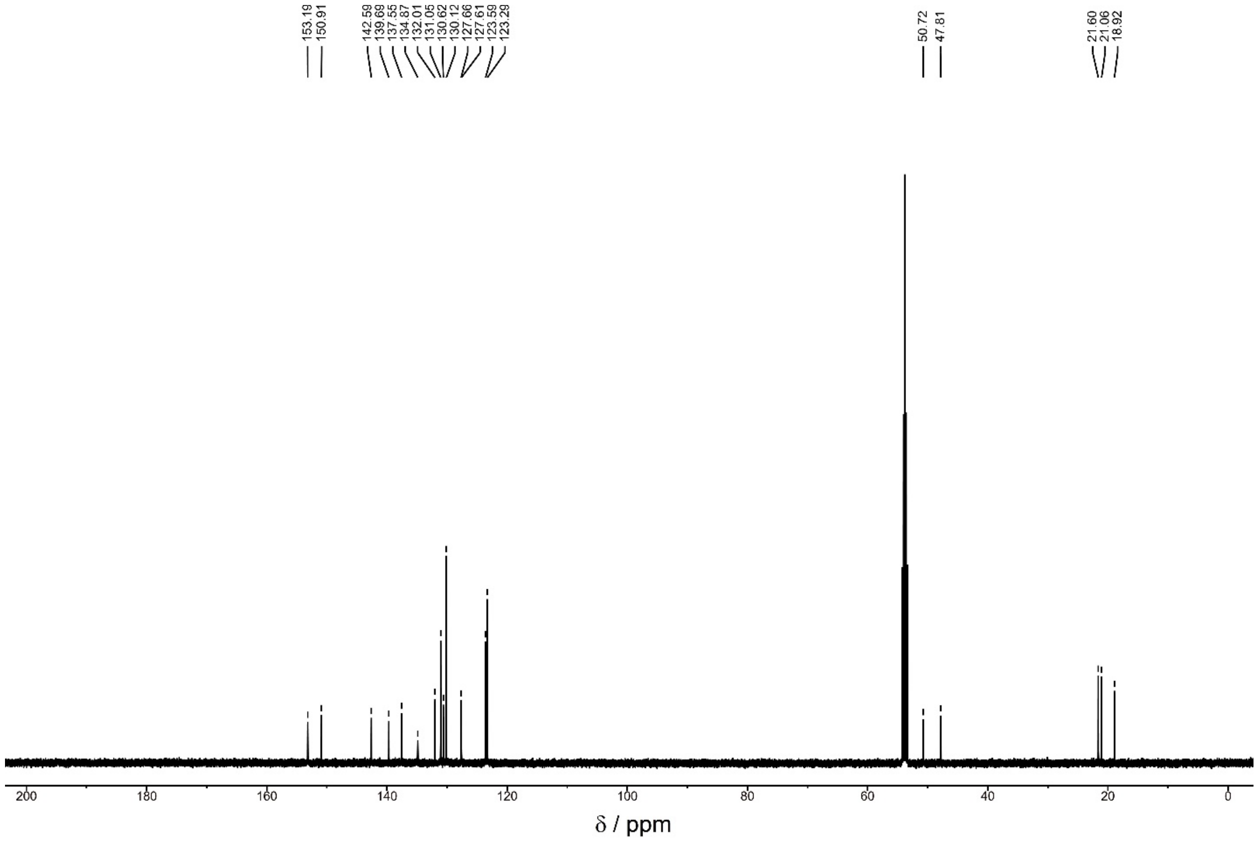

Figure S2: ^{13}C NMR Spectrum (500 MHz, CD_2Cl_2, 298 K) of compound *E*-**3**$^+$.

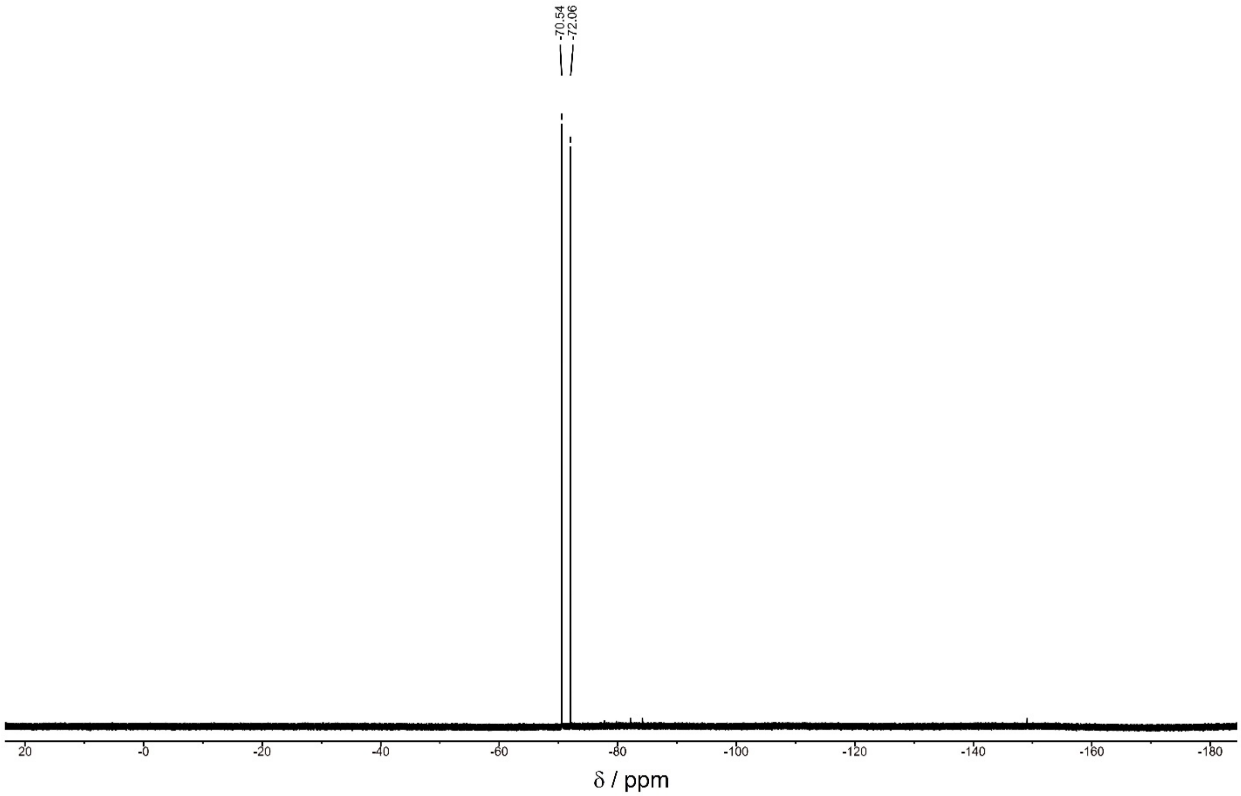

Figure S3: ^{19}F NMR Spectrum (500 MHz, CD_2Cl_2, 298 K) of compound *E*-**3**$^{+}$.

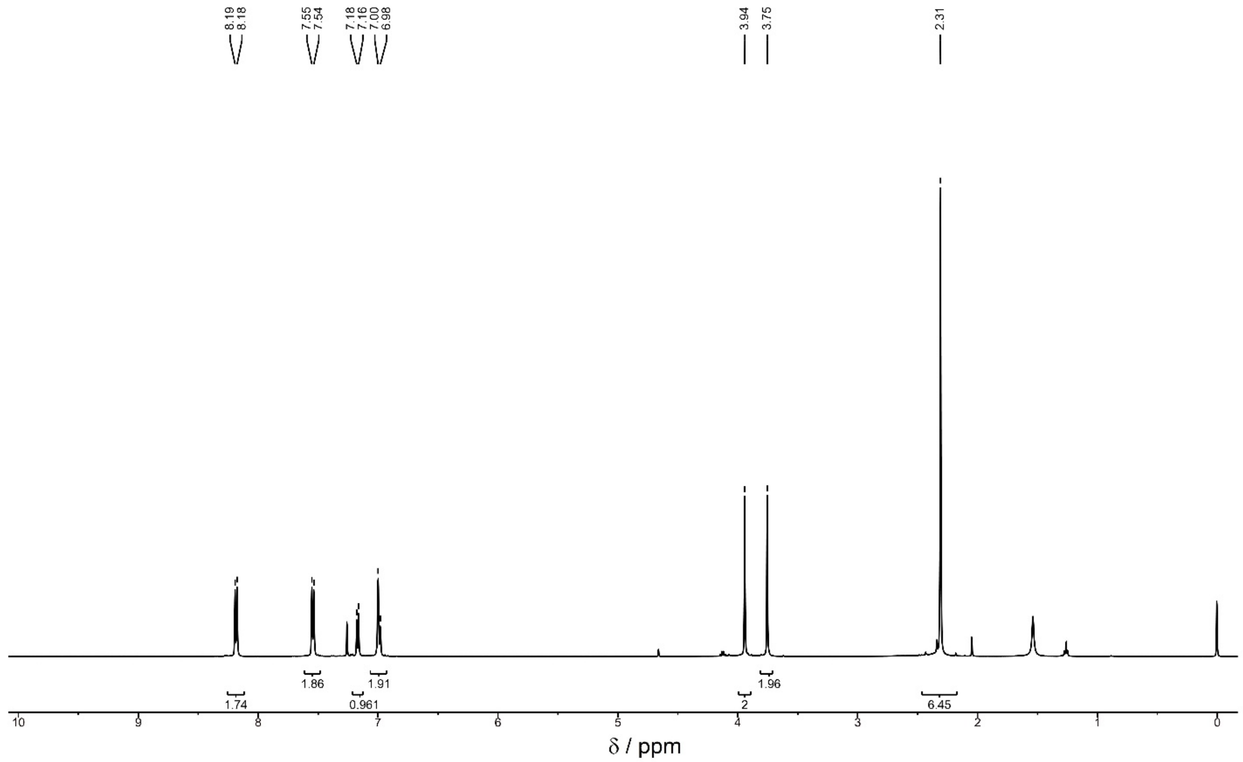

Figure S4: ^{1}H NMR Spectrum (500 MHz, CD_2Cl_2, 298 K) of compound **6**.

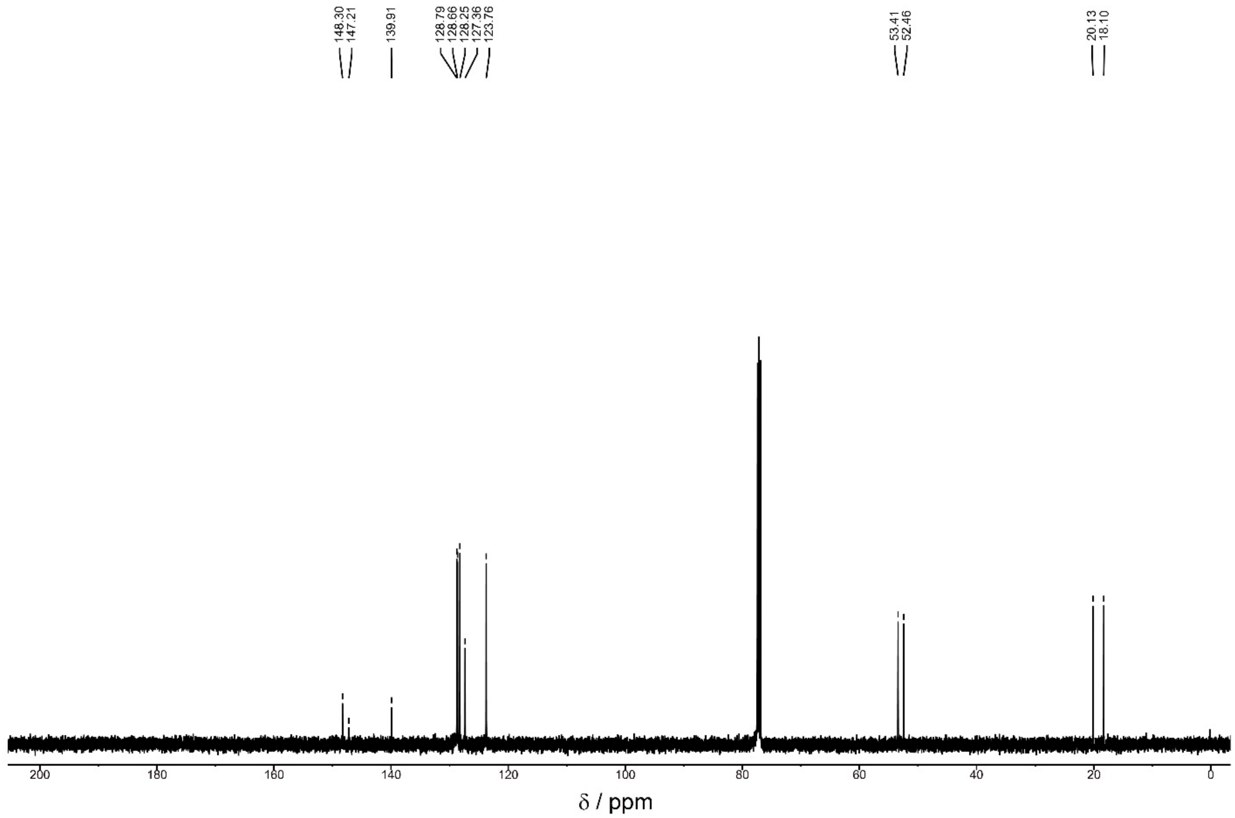

Figure S5: ^{13}C NMR Spectrum (500 MHz, CD_2Cl_2, 298 K) of compound **6**.

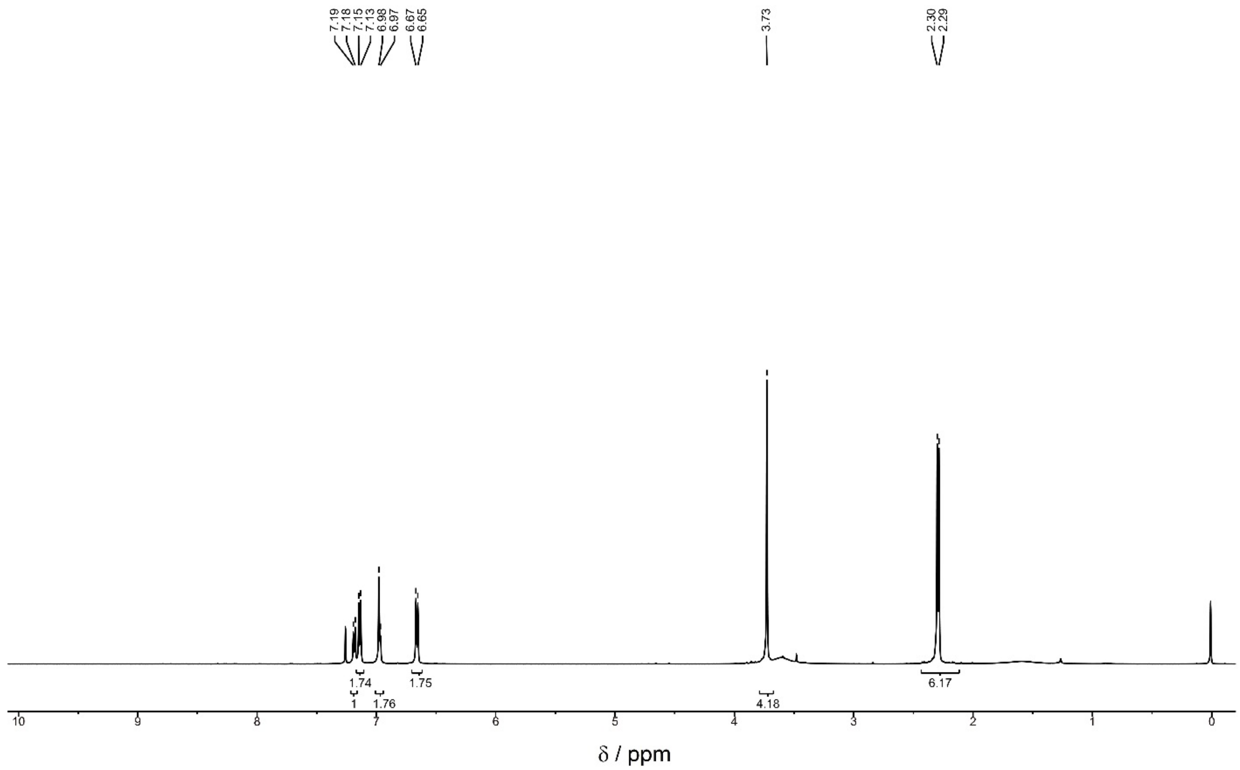

Figure S6: ^{1}H NMR Spectrum (500 MHz, CD_2Cl_2, 298 K) of compound **7**.

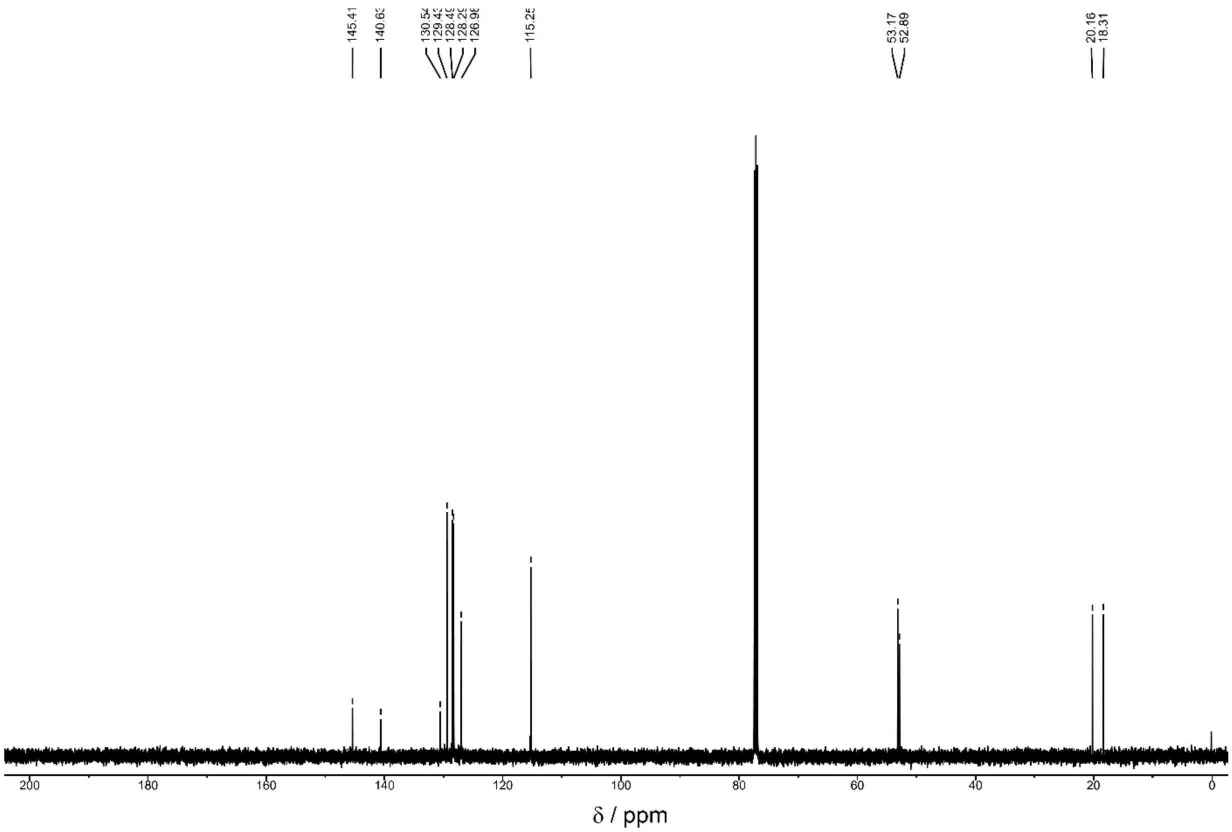

Figure S7: ^{13}C NMR Spectrum (500 MHz, CD_2Cl_2, 298 K) of compound **7.**

3.2. ^{1}H NMR Spectra recorded in CD_2Cl_2 (Consistent with the UV-Vis measurements)

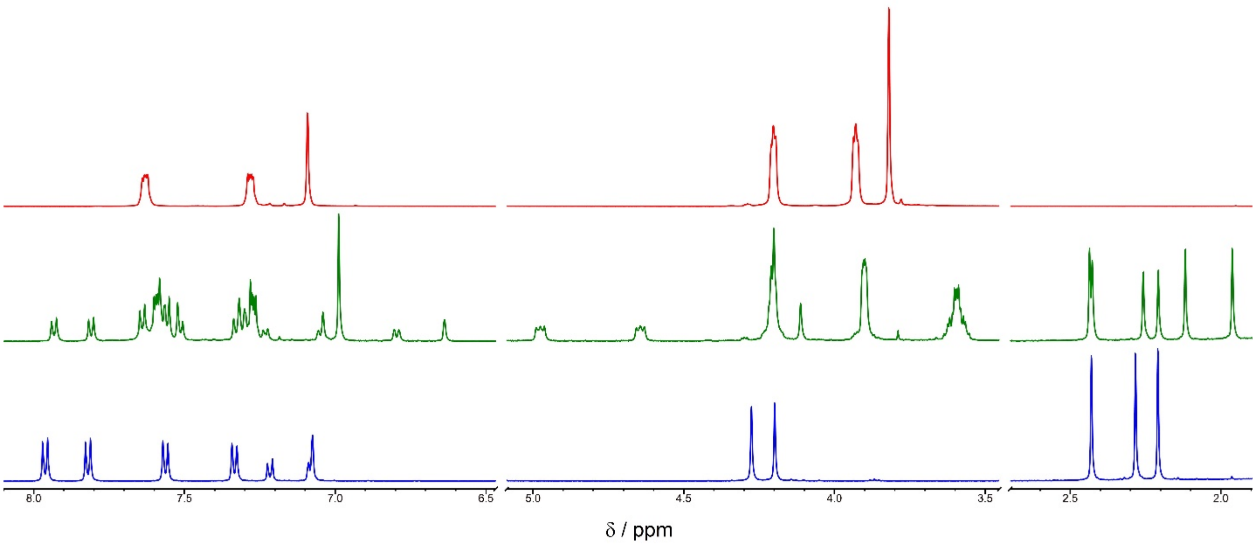

Figure S8: ^{1}H NMR Spectra (500 MHz, CD_2Cl_2, 298 K) of a solution of *E*-**3**$^+$ (5 mM) (blue line), an equimolar of *E*-**3**$^+$ and **1** (5 mM) (black line the signals associated with the complex are highlighted in green) and a solution of **1** (5 mM) (red line).

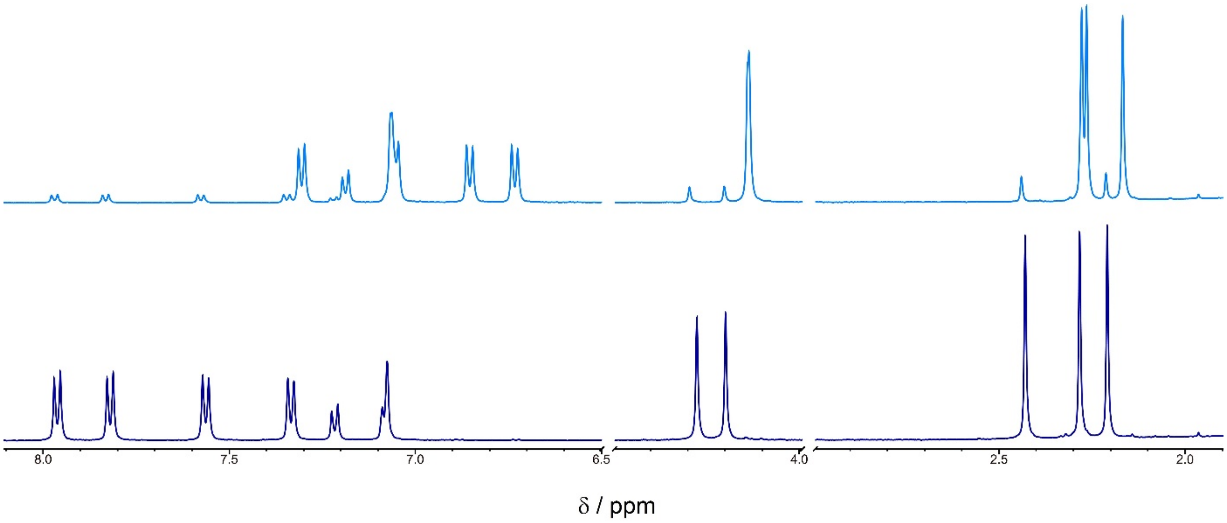

Figure S9: ^{1}H NMR Spectra (500 MHz, CD_2Cl_2, 298 K) of a solution of *E*-**3**$^+$ (5 mM) (dark blue line) and after irradiation at 365 nm (light blue line).

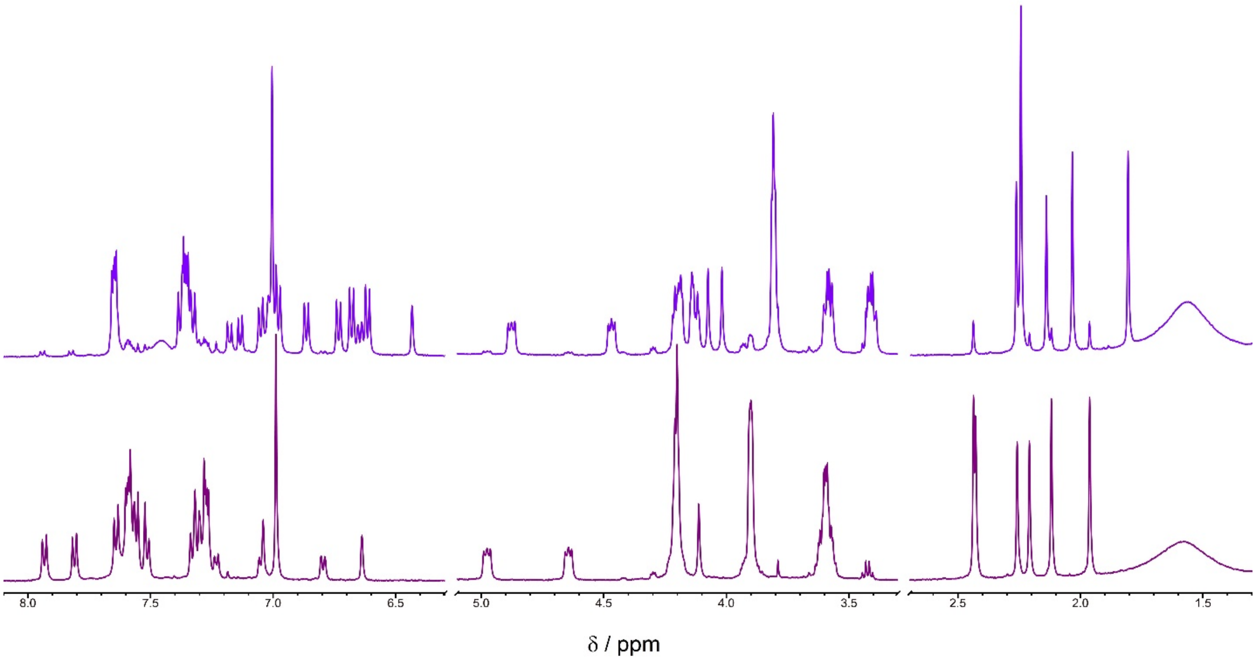

Figure S10: ^{1}H NMR Spectra (500 MHz, CD_2Cl_2, 298 K) of a solution of [*E*-**3**⊂**1**]$^{+}$ (5 mM) (dark purple line) and after irradiation at 365 nm (light purple line).

3.3. ^{1}H NMR Spectra recorded in CD_3CN/CD_2Cl_2 (1:1) solvent mixture (Consistent with the equilibrium and out of equilibrium NMR experiments)

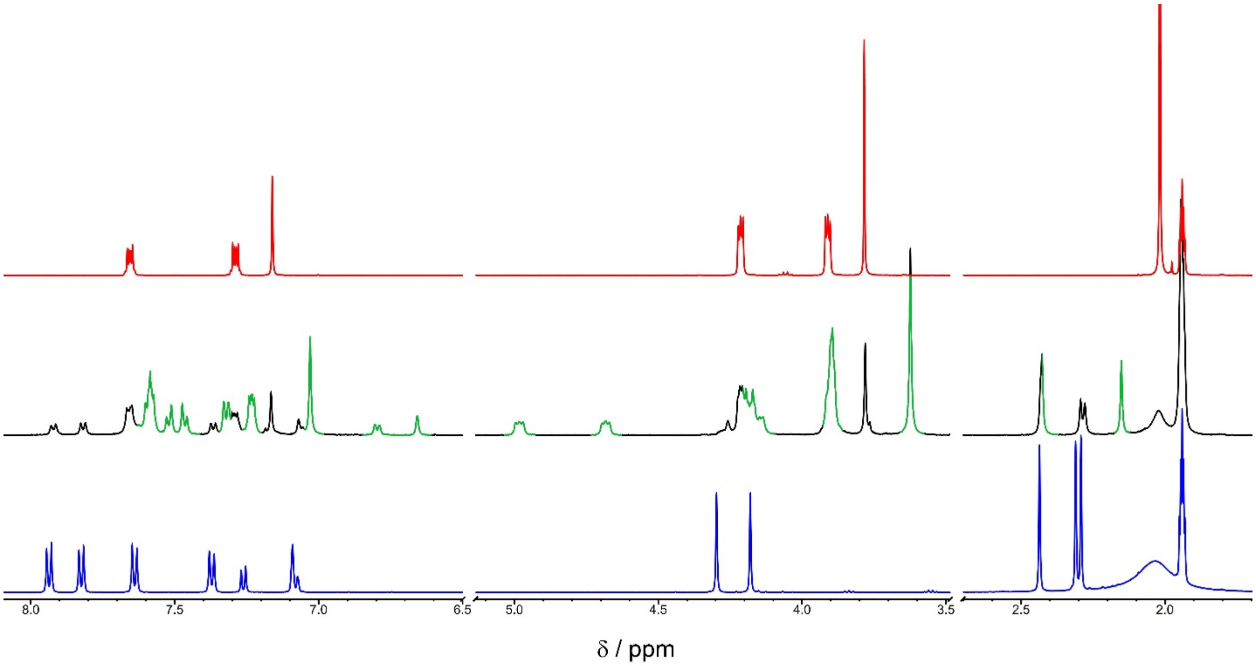

Figure S11: ^{1}H NMR Spectra (500 MHz, CD_3CN:CD_2Cl_2 1:1, 298 K) of a solution of *E*-**3**$^{+}$ (5 mM) (blue line), an equimolar of *E*-**3**$^{+}$ and **1** (5 mM) (black line, the signals associated with the complex are highlighted in green) and a solution of **1** (5 mM) (red line).

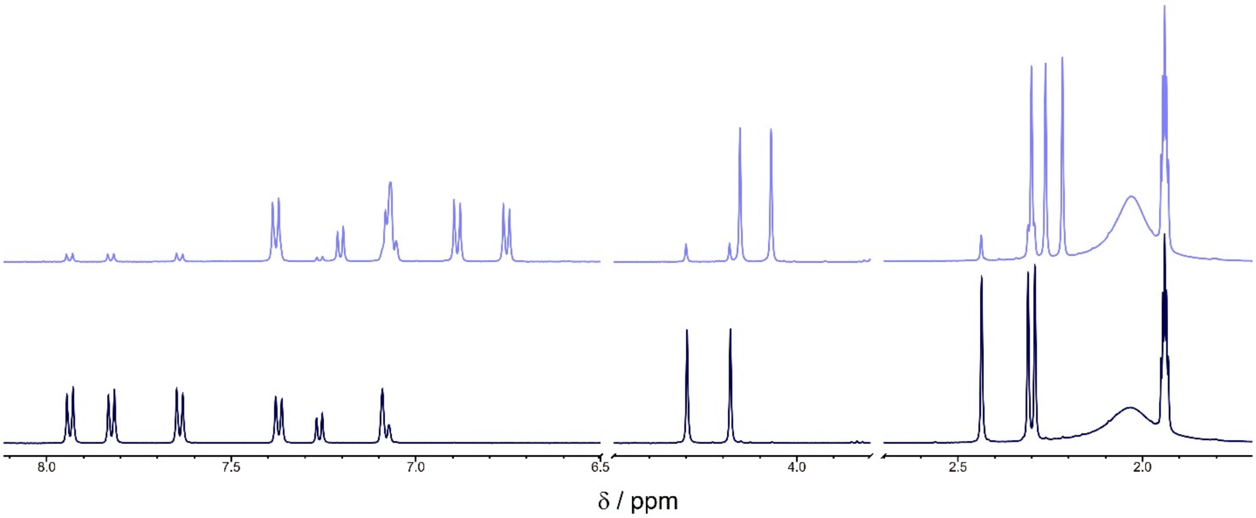

Figure S12: ^{1}H NMR Spectra (500 MHz, CD_3CN:CD_2Cl_2 1:1, 298 K) of a solution of *E*-**3**$^{+}$ (5mM) (black line) and after irradiation at 365 nm (light purple line).

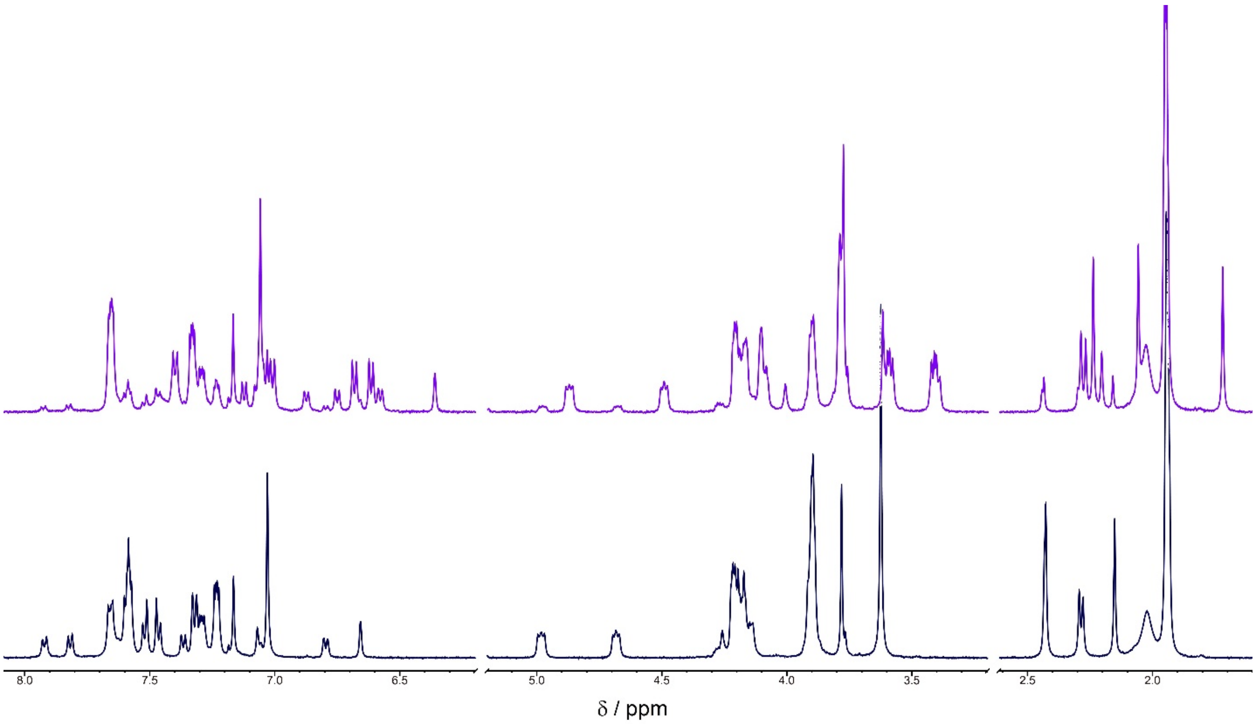

Figure S13: ^{1}H NMR Spectra (500 MHz, CD_3CN:CD_2Cl_2 1:1, 298 K) of a solution of [*E*-**3**⊂**1**]$^+$ (5 mM) (black line) and after irradiation at 365 nm (dark purple line).

4. UV-Visible Spectroscopy

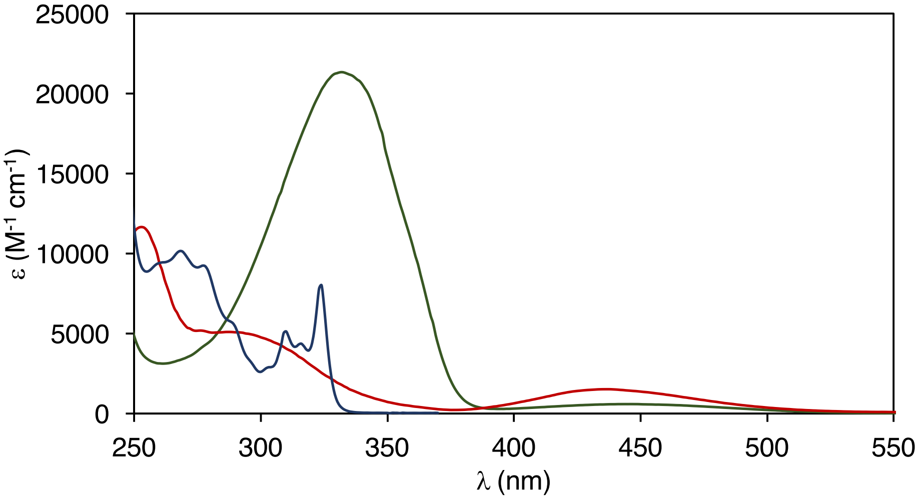

Figure S14: Absorption spectra (CH_2Cl_2, 293 K) of **1** (blue line), *E*-**3**$^+$ (green line), and *Z*-**3**$^+$ (red line).

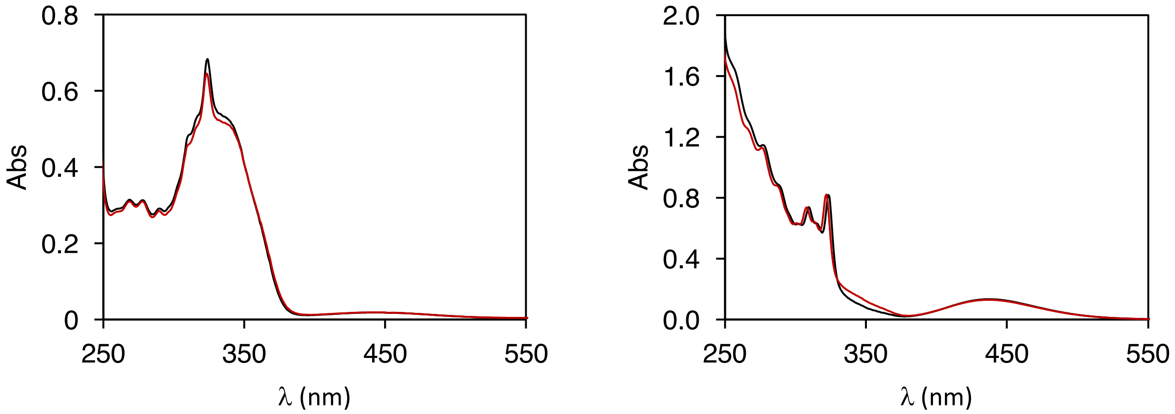

Figure S15: Sum of the absorption spectra (CH_2Cl_2, 293 K) of 86 μM **1** and *E*-**3**$^+$ (left, black line), and of 200 μM **1** and *Z*-**3**$^+$ (right, black line), and absorption spectrum of the mixture of the same two compounds (red line).

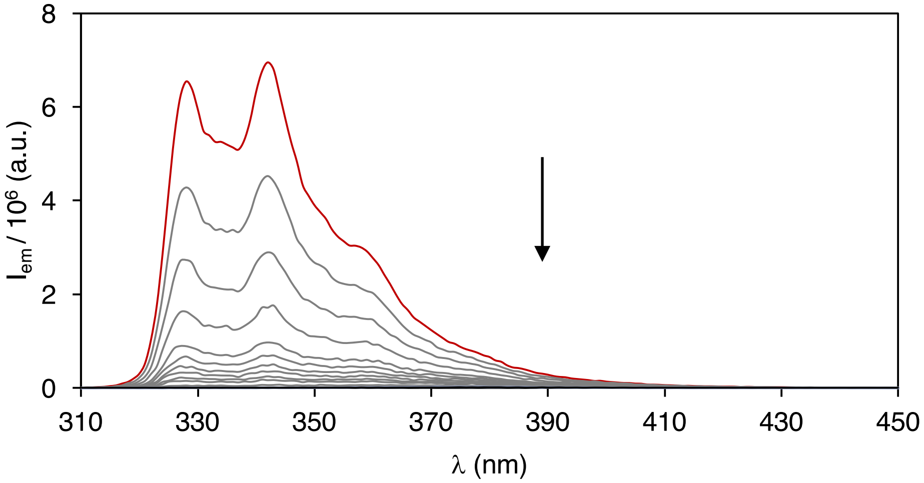

Figure S16: Emission spectra (λ_{exc} = 281 nm, CH_2Cl_2, 293 K) recorded upon titration of a 50 μM solution of **1** (red line) with small aliquots of *E*-$\mathbf{3}^+$. The corresponding titration curve is reported in Figure 2a in the main text.

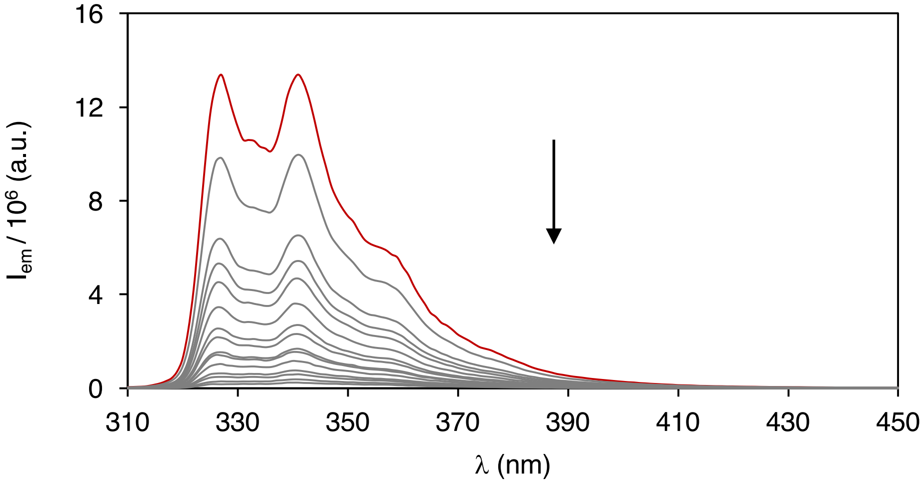

Figure S17: Emission spectra (λ_{exc} = 281 nm, CH_2Cl_2, 293 K) recorded upon titration of a 50 μM solution of **1** (red line) with small aliquots of *Z*-$\mathbf{3}^+$. The corresponding titration curve is reported in Figure 2b in the main text.

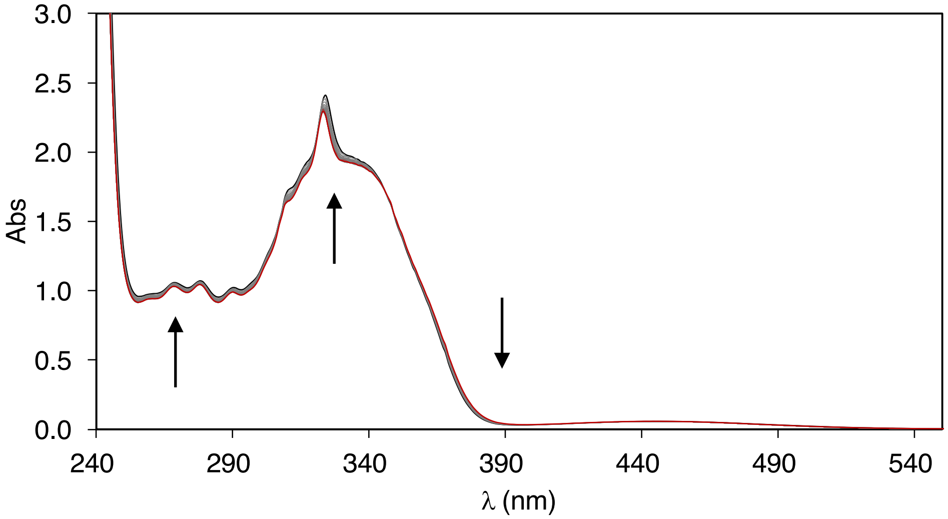

Figure S18: Time-dependent absorption spectra changes (CH_2Cl_2, 293 K) observed upon mixing 100 μM **1** with 100 μM *E*-**3**$^+$. The corresponding kinetic profile is reported in Figure 2a in the main text.

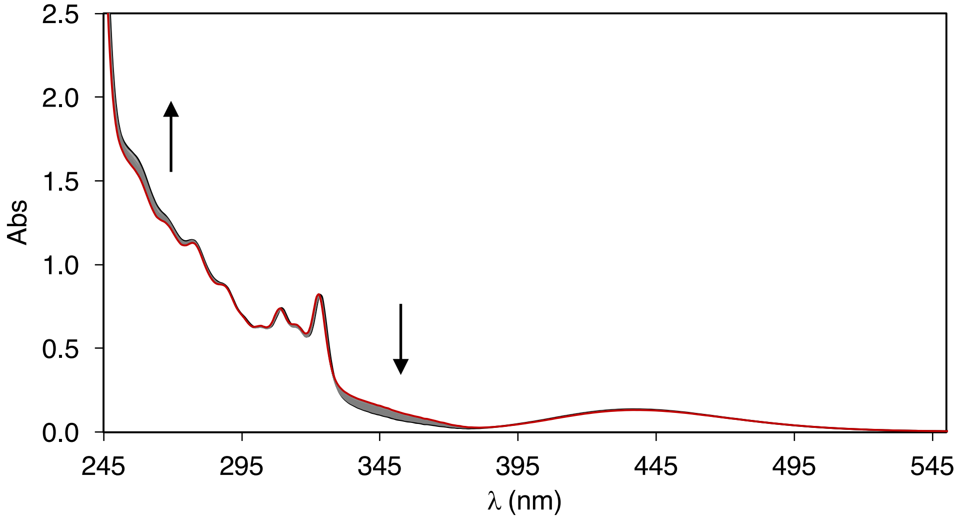

Figure S19: Time-dependent absorption spectra changes (CH_2Cl_2, 293 K) observed upon mixing 100 μM **1** with 100 μM *Z*-**3**$^+$. The corresponding kinetic profile is reported in Figure 2b in the main text.

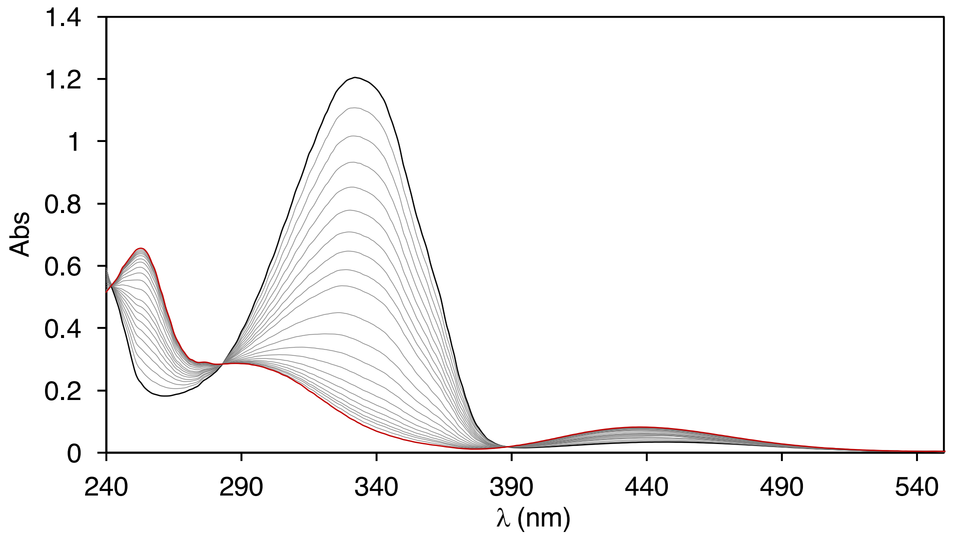

Figure S20: Absorption spectrum (CH_2Cl_2, 293 K) of 56 μM *E*-**3**$^+$ (black line), and spectral changes observed upon irradiation of the solution at 365 nm (grey lines), showing the occurrence of the *E*→*Z* photoisomerization. The red line is the absorption spectrum at the PSS (96% *Z* form), which can be practically taken as that of *Z*-**3**$^+$.

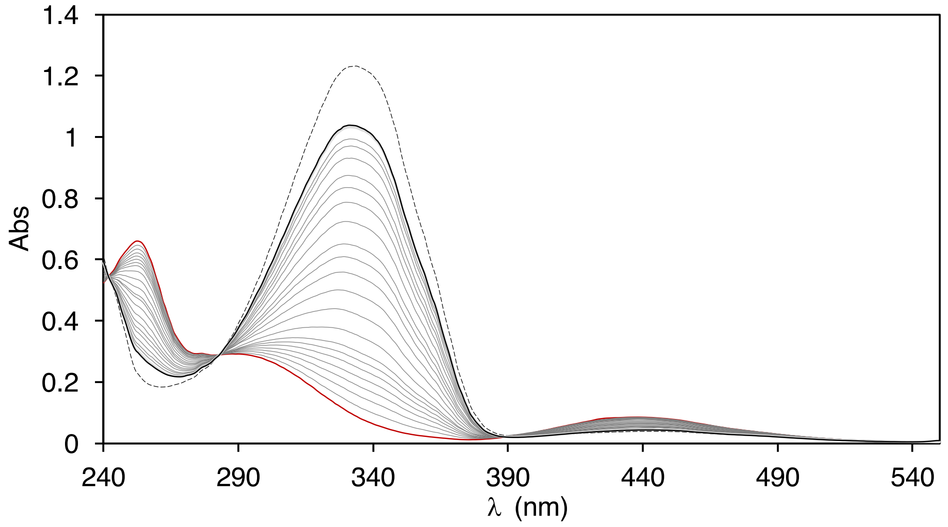

Figure S21: Absorption spectrum (CH_2Cl_2, 293 K) of 56 μM *Z*-**3**$^+$ (red line), and spectral changes observed upon irradiation of the solution at 436 nm (grey lines), showing the occurrence of the *Z*→*E* photoisomerization. The black line is the absorption spectrum observed at the PSS, while the dashed line shows the absorption spectrum of the initial *E*-**3**$^+$.

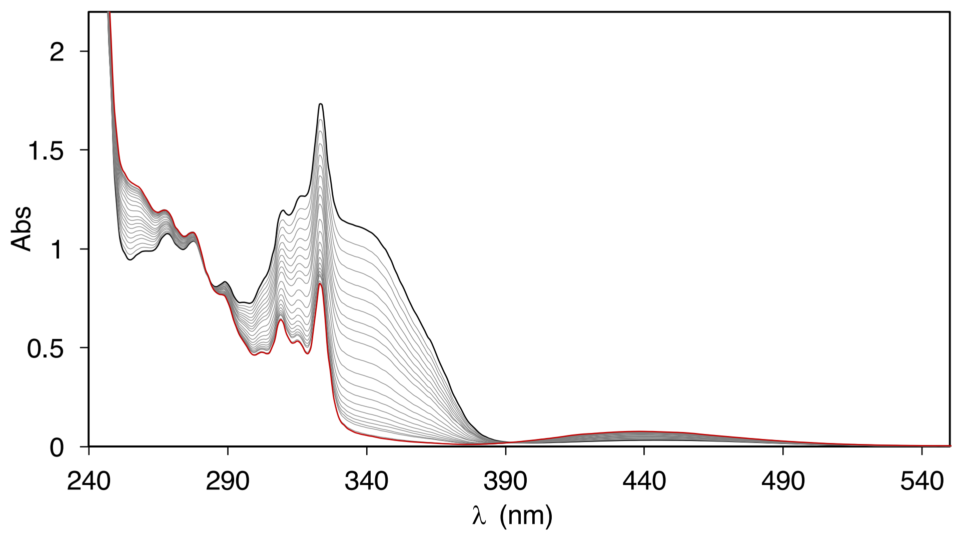

Figure S22: Absorption spectrum (CH_2Cl_2, 293 K) of 56 μM [*E*-**3**⊂**1**]$^+$ (black line), and spectral changes observed upon irradiation of the solution at 365 nm (grey lines), showing the occurrence of the *E*→*Z* photoisomerization. The red line is the absorption spectrum at the PSS (96% *Z* form), which can be practically taken as that of [*Z*-**3**⊂**1**]$^+$.

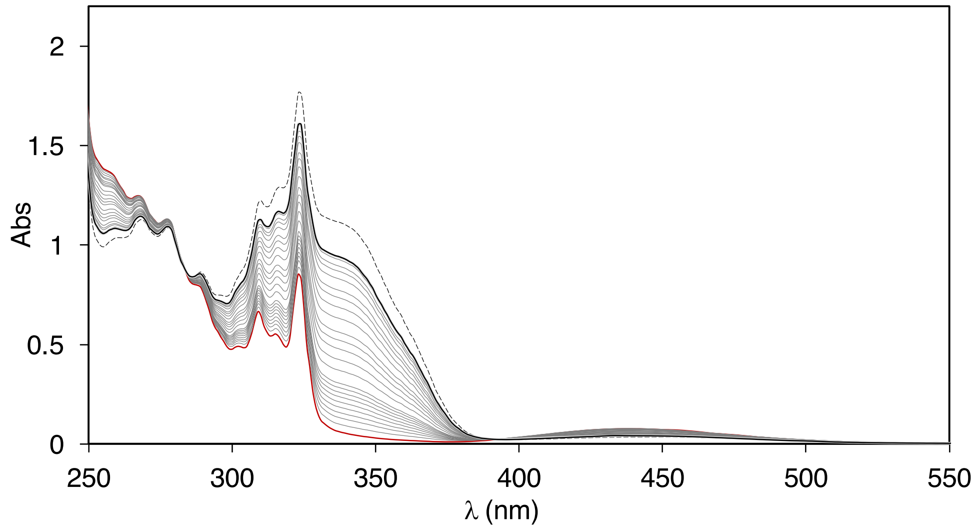

Figure S23: Absorption spectrum (CH_2Cl_2, 293 K) of 56 μM [*Z*-**3**⊂**1**]$^+$ (red line), and spectral changes observed upon irradiation of the solution at 436 nm, showing the occurrence of the *Z*→*E* photoisomerization. The black line is the absorption spectrum observed at the PSS, while the dashed line shows the absorption spectrum of the initial [*E*-**3**⊂**1**]$^+$.

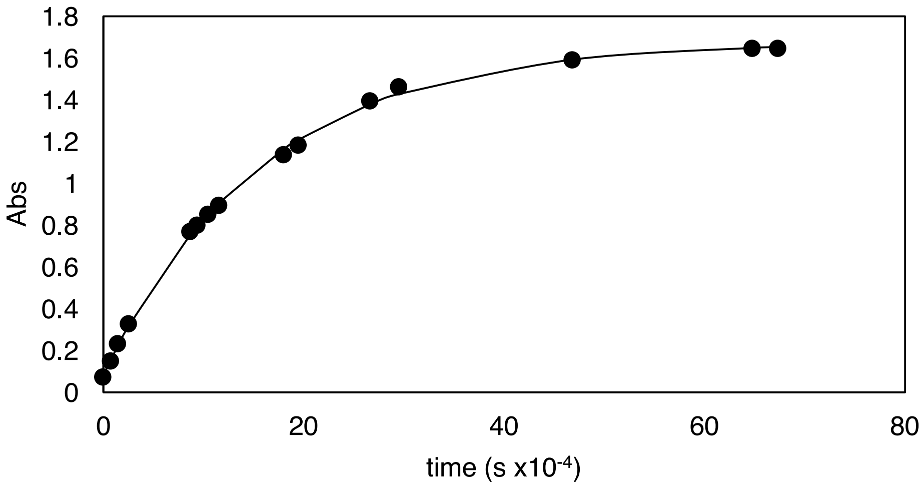

Figure S24: Time-dependent absorption changes at 350 nm observed on a 100 µM solution of *Z*-**3**$^+$ in the dark, highlighting the occurrence of the *Z*-**3**$^+$$\rightarrow$*E*-**3**$^+$ thermal isomerization. The line is the data fitting according to a first-order kinetic equation, corresponding to a rate constant $k_{Z\rightarrow E}$ = 6.3$\times$10^{-6} s^{-1} (CH_2Cl_2, 293 K).

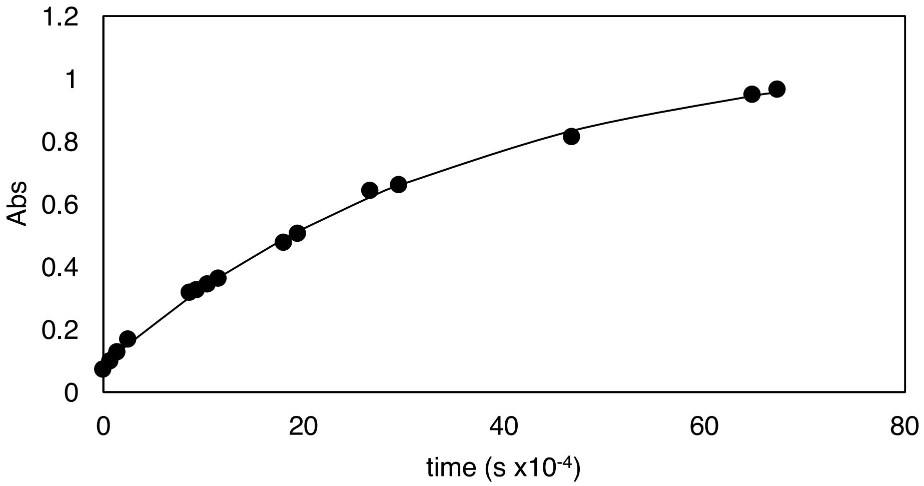

Figure S25: Time-dependent absorption changes at 350 nm observed on a 100 µM solution of [*Z*-**3**$\subset$**1**]$^+$ in the dark, highlighting the occurrence of the [*Z*-**3**$\subset$**1**]$^+$$\rightarrow$[*E*-**3**$\subset$**1**]$^+$ thermal isomerization. The line is the data fitting according to a first-order kinetic equation, corresponding to a kinetic constant $k_{Z\rightarrow E}$ = 3$\times$10^{-6} s^{-1} (CH_2Cl_2, 293 K).

Table S1. Photochemical and thermal isomerization data (CH_2Cl_2, 293 K).

Compound	$\Phi_{E\to Z}$ λ_{irr} = 365 nm	$[Z]/[E]_{PSS}$ λ_{irr} = 365 nm	$\Phi_{Z\to E}$ λ_{irr} = 436 nm	$[E]/[Z]_{PSS}$ λ_{irr} = 436 nm	$k_{Z\to E}$ (s^{-1})
**3**$^+$	0.17	96:4	0.65	84:16	6.3×10^{-6}
[**3**⊂**1**]$^+$	0.17	96:4	0.65	84:16	3.0×10^{-6}

Table S2. Free energy changes for the formation of the complexes (CH_2Cl_2, 293 K).

Complex	$-\Delta G°$ [a] kcal mol^{-1}	$\Delta G^‡_{in}$ [b] kcal mol^{-1}	$\Delta G^‡_{out}$ [b] kcal mol^{-1}
[*E*-**3**⊂**1**]$^+$	8.9	14.9	23.8
[*Z*-**3**⊂**1**]$^+$	7.3	16.1	23.5
[*E,E*-**4**⊂**1**]$^+$	> 9.4	14.8	> 24
[*Z,Z*-**4**⊂**1**]$^+$	—	19.0	—
[**5**⊂DB24C8]$^+$	7.1	16.0	23.1

[a] Standard free energies of association ($\Delta G°$), calculated from the *K* values (Table 1) using the expression $\Delta G° = -RT \ln K$. [b] Activation free energies for the threading ($\Delta G^‡_{in}$) and dethreading ($\Delta G^‡_{out}$) processes, calculated using the relationships $\Delta G^‡_{in} = -RT \ln(k_{in}h/kT)$ and $\Delta G^‡_{out} = -RT \ln(k_{out}h/kT)$.

5. References

(1) Sucholeiki, I. *J. Org. Chem.* **1988**, *1*, 98-104.

(2) Defoin, A. *Synthesis* **2004**, *5*, 706-710.

(3) Pedersen, C. J. *J. Am. Chem. Soc.* **1969**, *89*, 7017-7036.

(4) Baroncini, M.; Silvi, S.; Venturi, M.; Credi, A. *Chem. Eur. J.* **2010**, *16*, 11580-11587.

(5) Groppi, J.; Casimiro, L.; Canton, M.; Corra, S.; Jafari-Nasab, M.; Tabacchi, G.; Cavallo, L.; Baroncini, M.; Silvi, S.; Fois, E.; Credi A. *Angew. Chem. Int. Ed.* **2020**, *59*, 14825-14834.

(6) Binstead, R. A. *SPECFIT*; Spectrum Software Associates: Chapel Hill, 1996.

(7) Montalti, M.; Credi, A.; Prodi, L.; Gandolfi, M. T. *Handbook of Photochemistry – Third Edition*; CRC Press: Boca Raton, 2006.

(8) Feldmeier, C.; Bartling, H.; Riedle, E.; Gschwind, R. M. *J. Magn. Reson.* **2013**, *232*, 39-44.
